# First description of the life cycle of the jellyfish *Rhizostoma luteum* (Scyphozoa: Rhizostomeae)

**DOI:** 10.1371/journal.pone.0202093

**Published:** 2018-08-22

**Authors:** Karen Kienberger, Marta Riera-Buch, Alexandre M. Schönemann, Vanessa Bartsch, Roland Halbauer, Laura Prieto

**Affiliations:** 1 Group Ecosystem Oceanography, Department of Ecology and Coastal Management, Instituto de Ciencias Marinas de Andalucía (CSIC), Cádiz, Spain; 2 Vienna Zoo, Vienna, Austria; University of California Irvine, UNITED STATES

## Abstract

Jellyfish blooms are a significant environmental problem that is increasing and may be influenced by anthropocentric practices such as overfishing, pollution, eutrophication, translocation, climate change, and ocean acidification. Many jellyfish have unknown life cycles leading to these blooms. We describe for the first time, the life cycle of scyphozoan jellyfish *Rhizostoma luteum* from the planula to the young medusa stages, based on laboratory observations. We also provide a preliminary assessment of temperature related to life stages. Comparisons were made with early life history stages of its sibling species *Rhizostoma pulmo* and *Rhizostoma octopus*. The life cycle of *R*. *luteum* follows the general pattern of metagenesis of scyphozoans. Scyphistoma culture was maintained in filtered seawater at 17–17.5 °C, salinity 37 and light photoperiod (12:12 h light:dark). Scyphistomae were exposed to an experimental temperature descent for two days to test their survival capacity under severe winter conditions. Only one asexual reproduction mode was observed, which is employed for propagation, consisting of podocyst formation with excystment, subsequent development of scyphistoma, strobilation and liberation of viable ephyra. The development of the ephyra to metaephyra was photodocumented, reaching the metaephyra stage in approximately 21–25 days. Young medusae grow rapidly and maturity was reached after a 3-month post-liberation period with a mean bell diameter of 13.27 ± 2.26 cm and wet weight of 181.53 ± 53 g. The life cycle of *R*. *luteum* resembles that of its congeners, with the distinction that it has the unique features of being a brooding species (internal fertilisation with subsequent release of planulae) and under the conditions tested, the predominantly strobilation type observed was monodisc, and not polydisc as with the other two species in the genus *Rhizostoma*. As *R*. *luteum* shows sufficient requisite to form blooms if environmental circumstances change, it is important to understand its life cycle.

## Introduction

In recent decades, blooms (sudden outbreaks of one species which comes to dominate the plankton for a period) of jellyfish in coastal waters appear to be increasing in both frequency and intensity, producing negative ecological, social, and economic impacts. Jellyfish blooms are a significant environmental problem and may be influenced by anthropocentric practices/stressors such as overfishing of predators and competitors, accidental translocations, eutrophication of coastal waters, pollution, aquaculture, artificial structures, changes in freshwater flows, ocean acidification, and climate change [1–5].

The scyphozoan medusa *Rhizostoma luteum* (Quoy & Gaimard 1827) is an eastern Atlantic species with a distribution range from Lisbon (39°N) to Angola (16°S), entering the Alboran Sea (southwestern Mediterranean Sea) through the Strait of Gibraltar with its most eastern range being 2°E (Aguilas, Cartagena) ([6, 7] and references therein). The northern Alboran Sea includes the world-renowned Costa del Sol, which counts for 42% of all tourism in Andalusia (southern Spain). The most important sector for Andalusia is the service sector (sea-based economies), with nearly 30 million annual visitors and with revenues from tourism of €13,830 million for 2017 [8]. Jellyfish stings are among the most common reasons for requesting medical assistance at first aid stations in the summer months. Negative media reports may arise if the closing of beaches persists because of the presence of jellyfish, and could drive tourists to seek alternative destinations. *Rhizostoma luteum* is moderately venomous in a similar manner to *R*. *pulmo* (Kienberger, pers. observ.). However, because of its larger size (about 70 cm in diameter [6, 7]), the presence of *Rhizostoma luteum* near the coasts can have a negative impact on the image of touristic areas, as aforementioned, which are very sensitive to such issues.

Rhizostomeae are characterised by the absence of marginal tentacles and the manubrium forms eight oral arms with numerous mouth openings [9, 10]. The genus *Rhizostoma* contains currently three species recognised as valid [6, 9, 10]: *Rhizostoma pulmo* (Macri 1778) from the Mediterranean Sea and the Black Sea; and *Rhizostoma octopus* (Linnaeus 1788) appearing in northwest European coastal waters. In 2013, Prieto et al. reported the first record of *R*. *luteum* in the past 60 years and confirmed the validation of this species in the genus using molecular analysis that was performed on the mitochondrial cytochrome c oxidase I (COI) [6]. Until the study in 2017 [7], it was believed that this jellyfish had a sporadic occurrence, however, the authors demonstrated that this *Rhizostoma* was not as rare as was previously assumed. *Rhizostoma* was merely misidentified in the past along with its congener *R*. *pulmo* and another Rhizostomeae, *Catostylus tagi*, since there is an overlapping of their distributions with *R*. *luteum* in some regions.

Several previous authors have provided a description of adult specimens of *R*. *luteum* [6, 7, 9–14], however, the stages of the life history have never been reported until the present study. Meanwhile, the life stages of its congeners are better studied by various authors. Because of the increased interest in jellyfish for both environmental and socio-economic reasons, it is important to study scyphozoan life cycle and population dynamics, as these can provide important information with which to understand inter-annual fluctuations and which may also offer clues towards a better understanding of their role in marine ecosystems. However, the complete life cycle has been described for only a few Rhizostomeae (reviewed in [15]), and the knowledge of the sessile stages is still scant. Scyphistomae for the vast majority of species have never been found in their natural environment on account of their small size. Considering that the varieties of modes of asexual reproduction are more diverse than previously assumed, their ecological consequences have probably been underestimated [16, 17]. It is necessary to understand all stages of the life cycle, and not just the free-swimming medusa stage. Furthermore, as the ephyra stage of most species is very similar, it is important to include a detailed identification key when describing a new life cycle, which can be used to identify ephyrae, thus enabling the early detection of harmful jellyfish blooms in plankton samples. A study in 2010 established in the pattern of the gastric system, the only character that was constant in newly detached ephyra which was, therefore, the most reliable characteristic for identification [18].

Because of its broad distribution, the minimum sea surface temperature varies greatly in winter from 14 °C for Portugal, Malaga and Cartagena, to 25 °C for the Gulf of Guinea [19]. Therefore, all or some life stages might be exposed to substantial temperature changes whereby, on the one hand, there is exposure to seasonal temperature fluctuation and, on the other hand, there are oscillations due to strong regional coastal upwelling [20–22]. Many studies have shown that temperature changes (warming or cooling) affect the asexual reproduction and strobilation rates of the scyphistomae (reviewed in [23]).

The aim of the present paper is to describe for the first time the stages of the life cycle from planulae to young medusae of *R*. *luteum*, based on observations made on laboratory cultures and data collected *in situ*. Comparisons are made of its life cycle with its sibling species *R*. *pulmo* and *R*. *octopus*. Additionally, we conducted a preliminary assessment of the effects of temperature on the early life stages.

## Materials and methods

### Ethics statement

The jellyfish *Rhizostoma luteum* is not an endangered or protected species. No specific permissions were required for the location and activities of our field studies since it is a public dive location.

### Planulae

**Fig 1:**
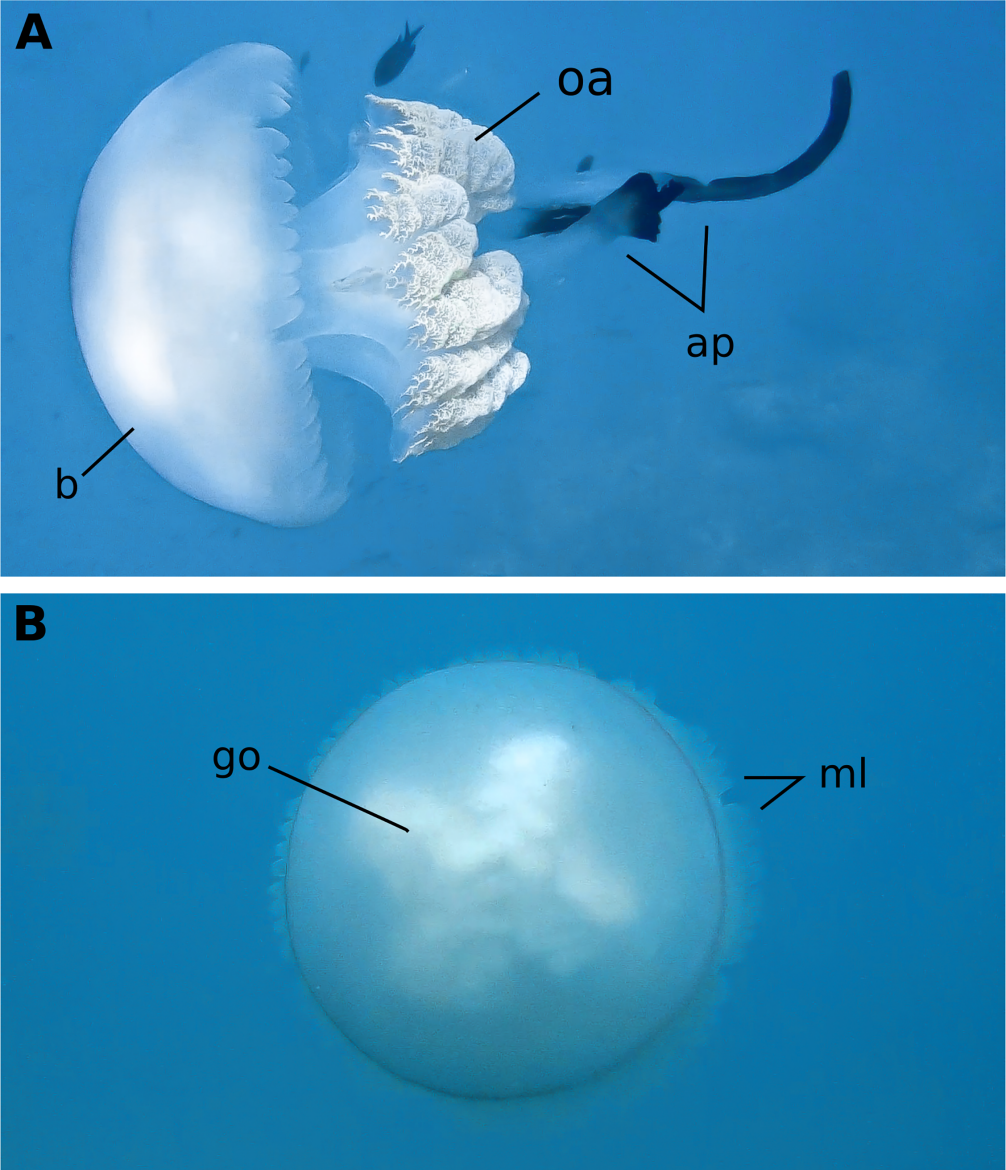
Adult *Rhizostoma luteum*. Female adult medusa swimming in the coastal water of La Herradura, NE Alboran Sea, photographed on 12th October 2015, bell diameter of 58 cm; **(A)** Side view; **(B)** Aboral view. *ap* appendages, *b* bell, *go* gonad, *ml* marginal lappet, *oa* oral arms. Photos by D. Enayati.

Planulae were collected on October 12, 2015, during weekly snorkel surveys and records were kept of the presence or absence of gelatinous organisms in the dive location, known as Marina del Este, La Herradura, southern Spain (36.720278° N, 3.728333° W). Planulae were gathered directly from the gonad of a free-swimming female medusa (Fig 1) by puncturing the epidermis, using small scissors and a Pasteur pipette under the water. The medusa was swimming at a depth of 4 m; the bell diameter was 58 cm (measured during the collection of the planulae). The sea temperature and salinity 21.06°C and 37.46, respectively, were obtained by using a conductivity-temperature-depth instrument (CTD, NKE Instrumentation). Directly after collecting the planulae (during less than one hour), their identity was confirmed using a microscope and they were allocated to 220 ml (diameter 6.5 cm; depth 5 cm) glass jars filled with non-filtered seawater. The following day, they were transported for subsequent development of the life cycle to the Institute of Marine Sciences of Andalusia (Spanish acronym is ICMAN-CSIC) in Cadiz, southern Spain, and to the Vienna Zoo, Austria.

### Scyphistomae

A glass slide (7.5 x 2.5 cm) was used as a substrate for the planulae to attach themselves to in each flask. The scyphistomae culture was maintained with filtered seawater without circulation in an incubator (IBERCEX F-4) with light photoperiod (12:12 h light:dark) and fed once a week with rotifers *at libitum* in the dark. Two hours after feeding, the rearing medium was exchanged with previously aerated seawater. The conditions of the seawater were 17–17.5 °C, salinity 37. Light intensity was 360 mmol quanta m^-2^ s^-1^. The light source consisted of four Philips MASTER TL-D 18W/840 fluorescent tubes. The light intensity was measured using a calibrated Biospherical Instrument sensor (QSL2100, San Diego, CA, USA).

Since *R*. *luteum* is common to the southwestern Mediterranean Sea, it is plausible that some medusae could eventually enter the coastal lagoon of the Mar Menor (southeast Spain). Due to the dimensions of the lagoon, the temperature can drop to 4 °C in severe winter conditions, which have been demonstrated to affect scyphistomae survival of other species [24]. In order to check the effect of a drop in temperature for this species, the experiment consisted of two treatments. Scyphistomae in the first treatment (4 replicates) were exposed to a temperature of 4 °C for two days, and the control group (3 replicates) stayed at a constant temperature. The average number on individuals per replicate was 6 (S2 Dataset). The drop in temperature was obtained by decreasing at a rate of 1 °C per hour. After two days at 4 °C, the scyphistomae were again maintained at the previously described culture conditions. The number of podocysts produced, podocysts developing into scyphistomae, and ephyrae liberated under constant condition were monitored weekly for 16 weeks. All stages were examined and photographed under a binocular dissecting microscope.

### Ephyrae

Ephyrae were cultured in 200-ml glass flasks at constant temperature and salinity, 23 °C and 33, respectively, and with light photoperiod (12:12) at the Aquarium House of the Vienna Zoo, Austria. Water baths maintained stable temperatures (± 0.5 °C) and artificial seawater was made by dissolving a commercially prepared sea salt mix (Instant Ocean^®^ Synthetic Sea Salts) in reverse-osmosis water waiting, 24 hours for the salt to dissolve properly. Ephyrae were fed daily with newly hatched *Artemia salina* nauplii, enriched with Selco^®^ S.presso *ad libitum*. After 4 hours of feeding, ephyrae were transferred daily, using a small container, to new flasks with fresh, previously aerated seawater.

Metaephyrae were reared to young medusae in laboratories at the Aquarium House of the Vienna Zoo, Austria in jellyfish kreisel tanks (hereinafter, kreisel) having volumes of 8, 15, 300 and 600 l. The 8 and 15 l kreisels were aerated but not connected to a Life Support System, hence 100% water changes were performed daily while the metaephyrae were fed twice daily with enriched *Artemia* nauplii. The kreisels were surrounded by a water bath, which was connected to an aquarium chiller (Aqua Medic Titan 1500). The temperature and salinity was at a constant, 24 ± 0.5 °C and 34 ± 0.5, respectively. Young medusae were transferred to kreisels with 300 and 600 l volume. Every kreisel was connected to a Life Support system consisting of a bio filter (10 l Sera Siporax), a protein skimmer (H&S 300-2xF5000) and an aquarium chiller (Aqua Medic Titan 1500). Freshly hatched enriched *Artemia salina* nauplii were given to the young medusae ad *libitum* twice daily. Water changes (100%) and basic cleaning of the kreisels were undertaken every 3 days.

Daily photographing of 10 ephyrae using a Nikon® SMZ800N stereomicroscope, and the image-processing NIS-Elements software, was undertaken in order to follow the development of the gastric system, manubrium and marginal lappets until reaching the metaephyra state (Stage 7). Each ephyra was placed in a petri dish with the manubrium facing upside. Magnesium was not used to achieve a slowed pulsation of the ephyra. Instead, the surrounding water was removed using a plastic pipette, leaving only a small sheet of water over the flattened ephyra. To avoid stress, the photo was taken as quickly as possible, when the ephyra was fully expanded. Afterwards, the medusa was transferred back into the culturing flask. The following standard measurements for scyphistomae [25] and ephyrae [18] were used: total body length (TBL), hypostome length (HL), calyx length (CL), stalk length (StL) and mouth disc diameter (MDD), total body diameter (TBD), central disc diameter (CDD), total marginal lappet length (TMLL), lappet stem length (LStL), rhopalial lappet length (RLL) and manubrium length (ML). We used the following proportions (%) to compare body proportions of scyphistomae compared to body length: CL/TBL x 100, HL/TBL x 100, StL /TBL x 100, MDD/TBL x 100. Moreover, ephyrae measurements compared to body diameter: RLL/TBD x 100, LStL/TBD x 100, CDD/TBD x 100 and lappet length: RLL/TMLL x 100, LStL/TMLL x 100. A total of 10 scyphistomae and 10 ephyrae from 10 strobilae were measured. Data are presented as mean ± 0.5 mm standard deviation.

### Young medusae

Twelve (12) young medusae reared in the laboratory were measured after 56 and 106 days post-liberation (n = 5 and n = 7, respectively) and compared to 7 young jellyfish that had been collected in their natural environment inside the marina (Puerto Deportivo Marina del Este, La Herradura, Southern Spain) in February 2016. During the sampling days, the sea temperature and salinity were obtained by using a conductivity-temperature-depth instrument (CTD, NKE Instrumentation) and had an average of 15.07 °C and 37.13, respectively. Individual bell diameter across the lappets was measured to the nearest 0.1 cm by placing the jellyfish flat onto a plastic ruler (oral side facing up), and it was weighed to the nearest 1 g. After amputating the manubrium at its base, morphometric measures were taken to the nearest 0.1 cm. Sex and gonadal maturity state were determined using a microscope.

### Statistics

Statistical analyses of the data were performed using R (v3.4.1) software. An ANOVA assumption was tested on a dataset prior to the evaluation of variance. If data failed normality, a non-parametric Kruskal-Wallis analysis of variance was used. To compare the data before and after the drop in temperature, a Welch Two Sample t-Test was conducted. The significance of which was judged at the 0.05 level. Linear regression was used to test the bell size variability in relation to the wet weight of a young medusa.

## Results

### Planulae

**Fig 2:**
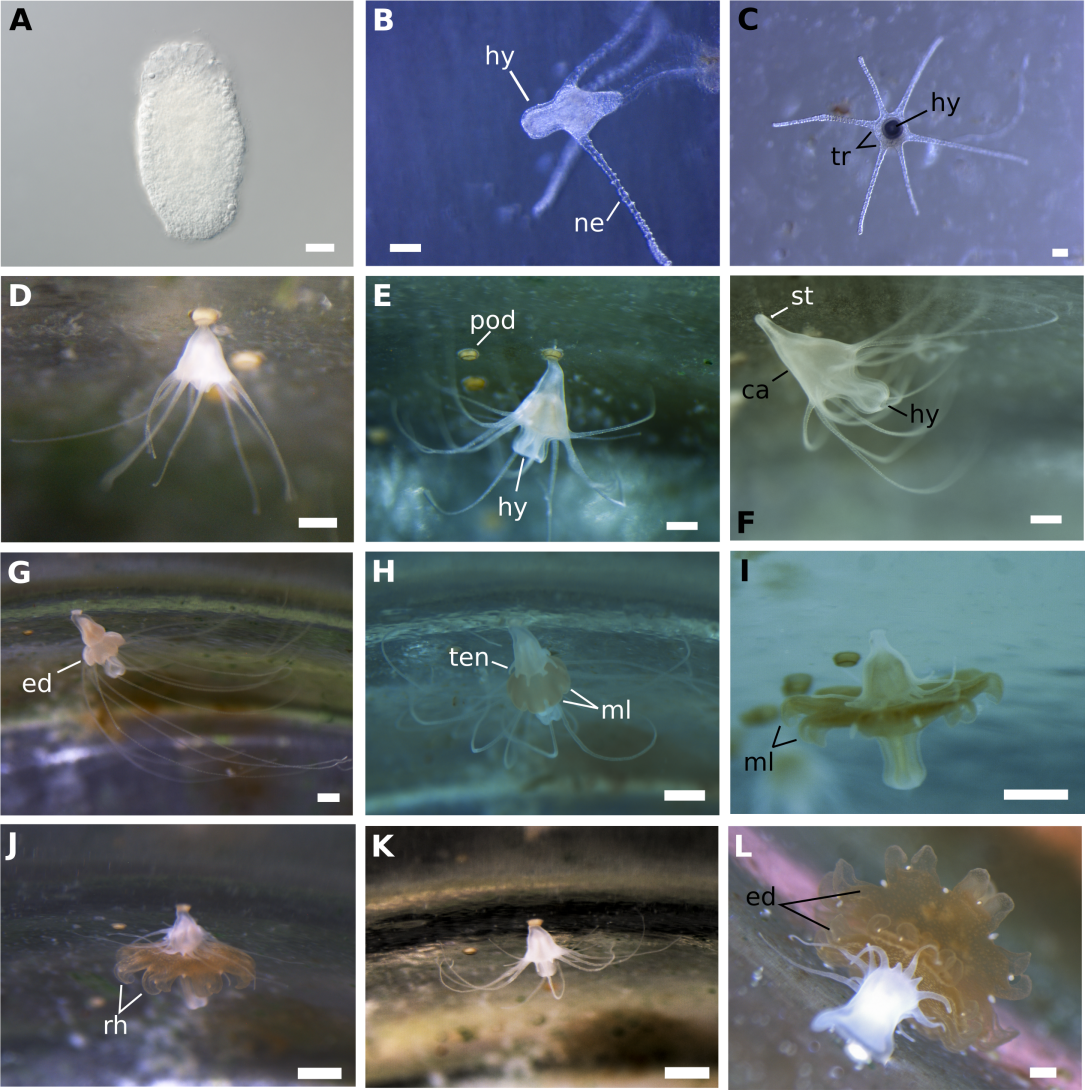
Various stages of the life cycle of *Rhizostoma luteum*. **(A)** Planula larva with a two-layered structure; **(B)** Newly metamorphosed 4-tentacled scyphistoma; **(C)** Top view of a 6-tentacled scyphistoma; **(D)** Young scyphistoma with 8 filiform tentacles and prominent hypostome; **(E)** Intermediate 12-tentacled scyphistoma; **(F)** Fully developed scyphistoma with 16 filiform tentacles; **(G-J)** Strobilation stages of the same scyphistoma during laboratory monitoring; **(G)** Early strobila with developing segments; **(H)** Basal scyphistoma regenerating new tentacles; **(I)** Mid-strobila with regressing tentacles and developing ephyral segment; **(J)** 4-day-old strobila about to liberate one ephyra; **(K)** Residuum after releasing ephyra; **(L)** Strobila with two ephyrae. *ca* calyx, *ed* ephyra disc, *hy* hypostome, *ml* marginal lappets, *ne* nematocysts batteries, *pod* podocyst, *rh* rhopalium, *st* stalk, *ten* tentacle, *tr* tentacular ring. Scale bar 1 mm for all except (A) 20 μm and (B, C) 100 μm. Photos by T. Schwaha: A; K. Kienberger: B, C; M. Riera-Buch and A.M. Schönemann: D-L.

**Fig 3:**
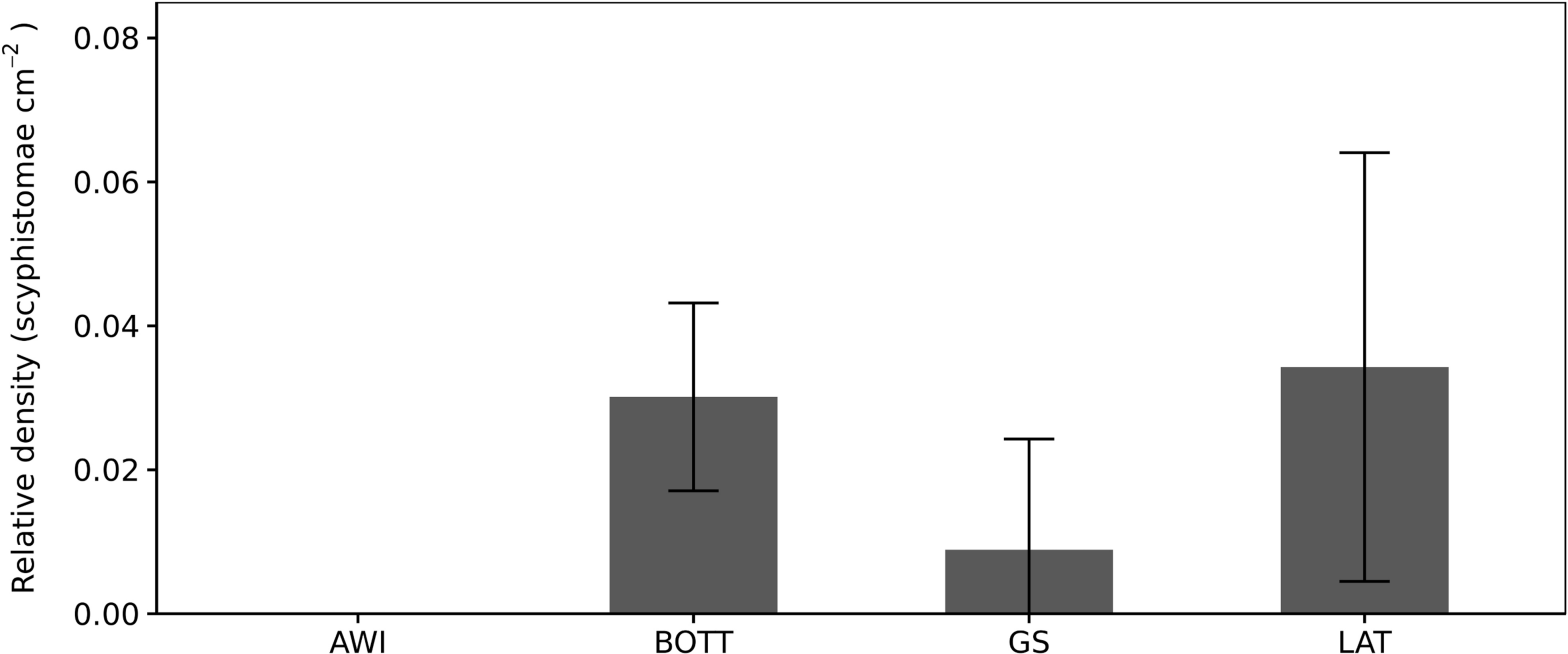
Settlement preferences of *Rhizostoma luteum* planulae. Mean density of scyphistomae per substrate. *AWI* air-water interface; *BOTT* bottom of the glass flasks: *GS* glass slides; *LAT* sides of glass flasks. Error bars are the standard deviations of the 4 replicates.

The gonads are visible from the aboral view in the adult jellyfish (Fig 1B). *Rhizostoma luteum* has separate sexes, and the planulae are brooded by the female medusae inside the gonadal cavity. Planulae had a two-layered structure, white in colour, oval and elongate (Fig 2A), the sizes varied from 126 to 139 μm in length and 73 to 106 μm in width (n = 4; 132 ± 5 μm lengths, 84 ± 15 μm widths). Planulae placed in glass jars immediately moved through the water column while rotating around their own axis, but then they were negatively buoyant, and accumulated near the bottom of the jar. Settlement of planulae was observed after 3 to 5 days; however, 15 days later some free-swimming planulae were still observed. There was no significant preference of settlement on the bottom of the glass flasks (BOTT), glass slide (GS) and sides of glass flasks (LAT) (Kruskal-Wallis test, P = 0.23; n = 20), see Fig 3. Notwithstanding, no settlement was observed at the air-water interface. The raw data file has been included in the Supporting Information (S1 Dataset).

### Scyphistomae

**Fig 4:**
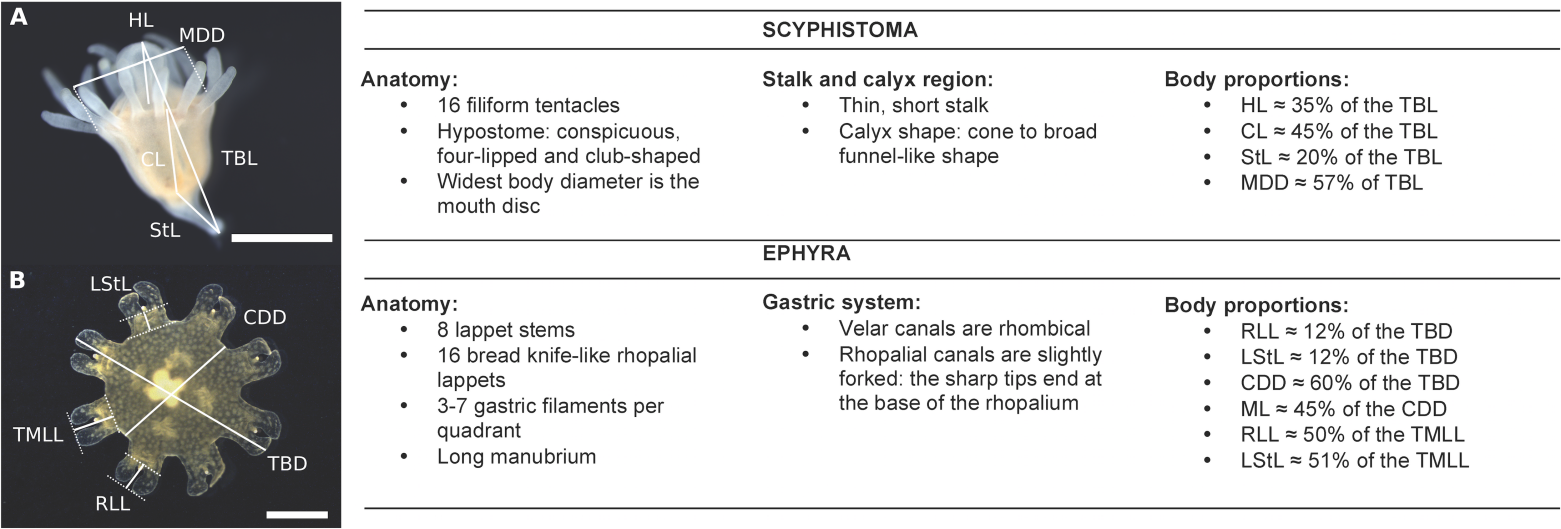
Morphology and body proportions of *Rhizostoma luteum* scyphistoma and ephyra. **(A)** Measuring points and measurements defined for scyphistomae: *CL* calyx length, *HL* hypostome length, *MDD* mouth disc diameter, *StL* stalk length, *TBL* total body length; **(B)** Measuring points and measurements for newly released ephyra: *CDD* central disc diameter, *LStL* stalk length, *ML* manubrium length, *RLL* rhopalial lappet length, *TBD* total body diameter, *TMLL* total marginal lappet length. Scale bar 1 mm. Photos by K. Kienberger.

**Fig 5:**
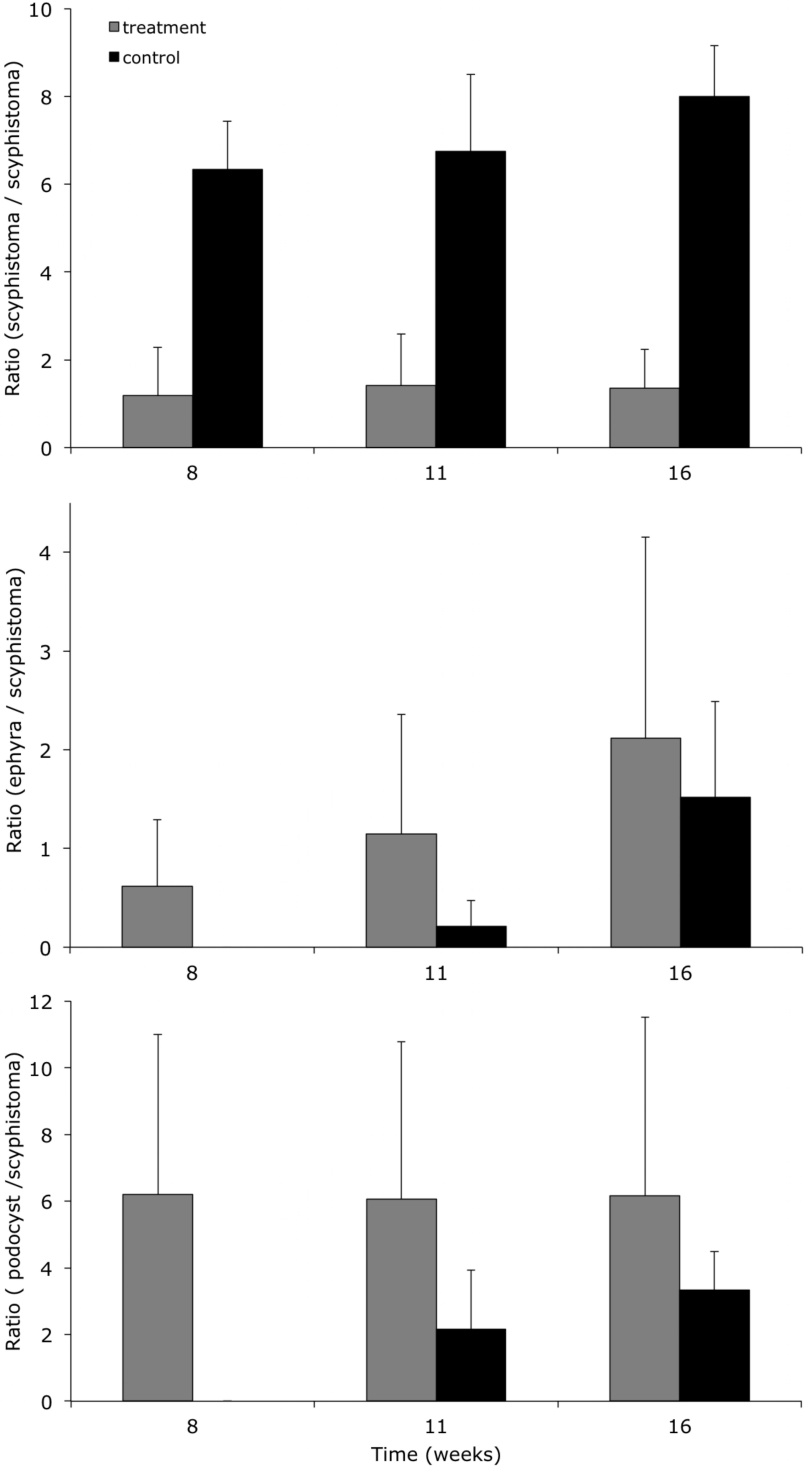
Production of *Rhizostoma luteum* scyphistoma, podocyst and ephyra. Average ratio per initial scyphistoma (n = 39) of the production of new scyphistoma, podocyst and ephyra after the scyphistomae were exposed to a drop in temperature and then a return to the regular temperature conditions (grey) and control (black). Error bars are the standard deviations of the 3–4 replicates.

Approximately 72 hours after the planulae had settled, translucent-white scyphistomae with four primary tentacles developed. Nematocysts batteries were visible on the tentacles (Fig 2B). Cup-shaped scyphistoma with 8 and 12 tentacles with a prominent club-shaped hypostome developed after approx. 2–3 weeks (Fig 2D and 2E). Around 5 weeks after the planulae had settled, fully developed (16-tentacles) scyphistomae matured (Fig 2F) and had a conical to broad funnel-like shape. The total body length (TBL) varied from 1.26 to 2.89 mm (n = 10; 1.79 ± 0.48 mm) and had a mean mouth disc diameter (MDD) width of 1.02 mm (see Fig 4 for body proportions). The number of tentacles was highly variable, up to 21, but typically scyphistomae had 16 filiform tentacles. A single whorl of tentacles surrounded the peristome and the conspicuous, four-lipped and club-shaped hypostome, about 35% of the TBL. The scyphistomae colour ranged from opaque to white and became light rose-orange after ingestion of nauplii. The base of the scyphistomae was attached to the substrate by a thin, short stalk (about 20% of TBL). Over the 16 weeks of monitoring after the decrease in temperature, ratios were calculated from the initial number of scyphistoma before the change in temperature, maintaining the ratio over time for the treatment with the temperature shock, and with a slight increase in the number of scyphistoma for the control (Fig 5). An independent-sample t-test was conducted to compare the total number of scyphistoma with and without a temperature shock. There was no significant difference in the mean number of scyphistoma with and without a temperature shock (see Table 1). The raw data file has been included in the Supporting Information (S2 Dataset).

**Table 1:** *Rhizostoma luteum* statistical results. Results of the Welch Two Sample t-Test, testing for differences of production of scyphistoma, podocyst and ephyra among treatments with and without (control) a temperature drop, *P* < 0.05 is considered significantly different.

	Time (week)	t	df	P value
**Scyphistoma**	8	-0.366	4.931	NS
	11	-0.447	4.655	NS
	16	-0.832	3.139	NS
**Podocyst**	8	-2.875	3.000	P = 0.032
	11	-3.662	3.281	P = 0.015
	16	-2.839	2.000	NS
**Ephyra**	8	-1.502	3.000	NS
	11	-1.427	3.037	NS
	16	-1.105	3.017	NS

*NS* Not significant

### Strobilation

The calyx elongated at the first phase of strobilation and the new ephyra formed immediately below the tentacle crown (Fig 2G). The developing ephyra acquired mustard to dark gold colourations, tentacular lobes on the distal segment became more pronounced and the basal scyphistoma regenerated new tentacles (Fig 2H). The ephyra lappets elongated, rhopalia appeared and the manubrium lengthened. Scyphistoma tentacles on the ephyra began to undergo regression, contracting and expanding periodically, until they were resorbed completely (Fig 2I and 2J). After liberation, the residuum TBL was equal as pre-strobilation and had already redeveloped 16 filiform tentacles (Fig 2K). At a constant temperature of 17–17.5 °C, the predominant type of strobilation monitored was monodisc (one ephyra per strobila), and during the entire experiment only once was a polydisc strobilation observed (Fig 2L). The duration of the strobilation process was 5–6 days and the mortality rate of scyphistomae after strobilation was nil. There was no significant difference in the total number of ephyra liberated between the two treatments (see Table 1). Both treatments increased ratio over time (see Fig 5). The raw data file has been included in the Supporting Information (S2 Dataset).

### Asexual reproduction of scyphistomae

**Fig 6:**
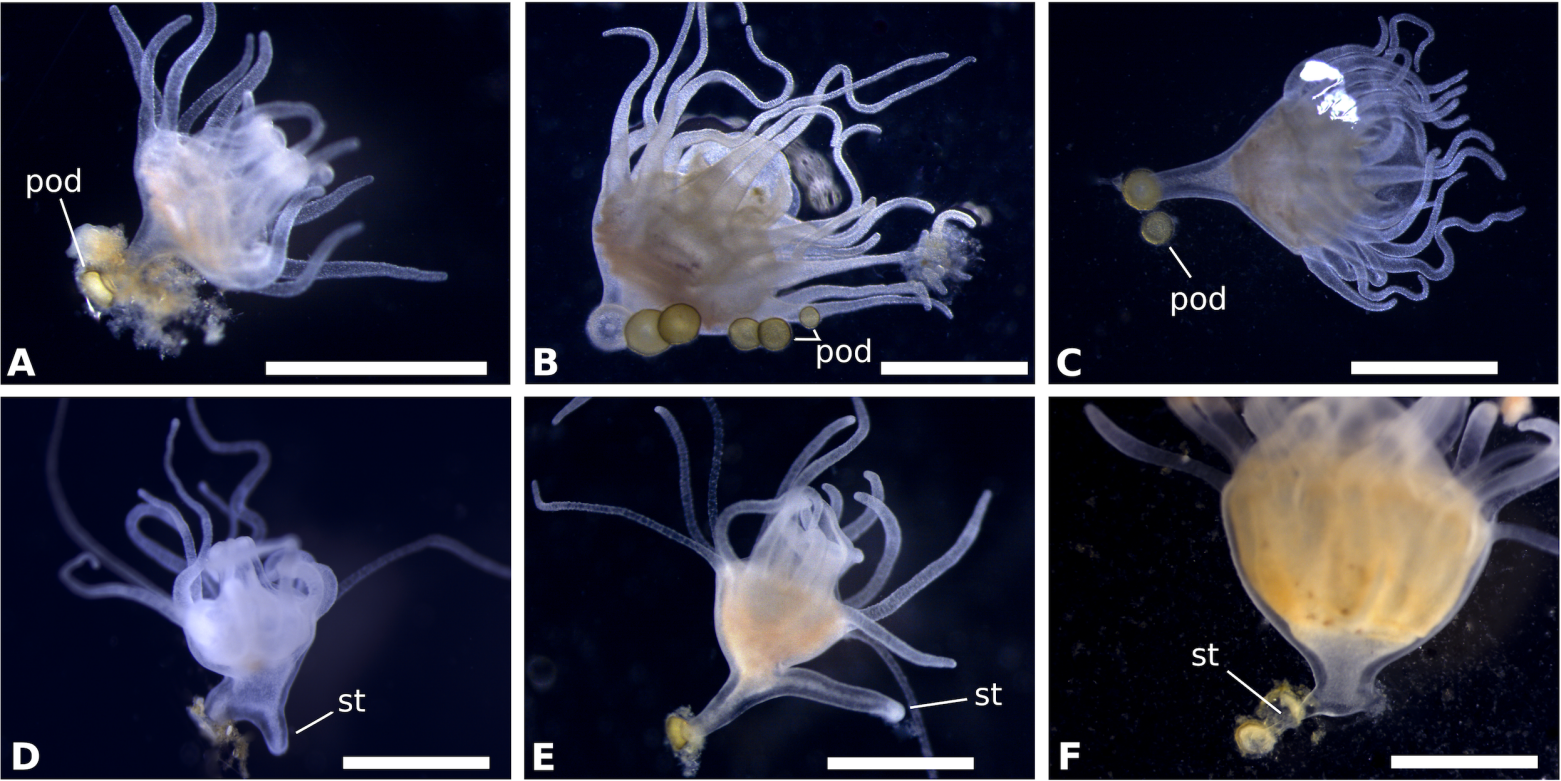
Asexual reproduction of *Rhizostoma luteum*: Podocyst formation by means of stolon. **(A)** Podocyst lateral view; **(B,C)** Formation of podocyst at the base of scyphistoma stalk; **(D-F)** Consecutive stolon development on the stalk. *pod* podocyst, *st* stolon. Scale bar 1 mm. Photos by K. Kienberger.

At the base of the stalk of fully developed scyphistomae (16-tentacles), podocyst formation (Fig 6) was observed by way of stolon formation. The podocyst were typically yellowish brown in colour (Fig 6A–6C), roundish discs with a concavity on top, the diameter ranged between 326 and 496 μm (n = 9; 430 ± 52 μm). Finger-shaped stolon developed from the lower stalk and attached to the substrate (Fig 6D–6F). It is noteworthy that this stolon never realised buds nor did it develop tentacles, as expected for lateral budding. Scyphistomae produce earlier podocyst in greater amounts and in the treatment using temperature shock (week 8) than without using temperature shock (week 11), see Fig 5. Mean podocyst production differed significantly by treatment according to an independent t-test only in week 8 and 11 (Table 1). The raw data file has been included in the Supporting Information (S2 Dataset). Furthermore, new scyphistomae excysted from the podocysts from the scyphistomae after 7 days with a temperature drop. Production increased over time, in the control treatment, but none of the scyphistomae excysted from the podocysts.

### Ephyrae

#### Stage 0: Newly-released ephyra from the strobila (Fig 7A–7D)

**Fig 7:**
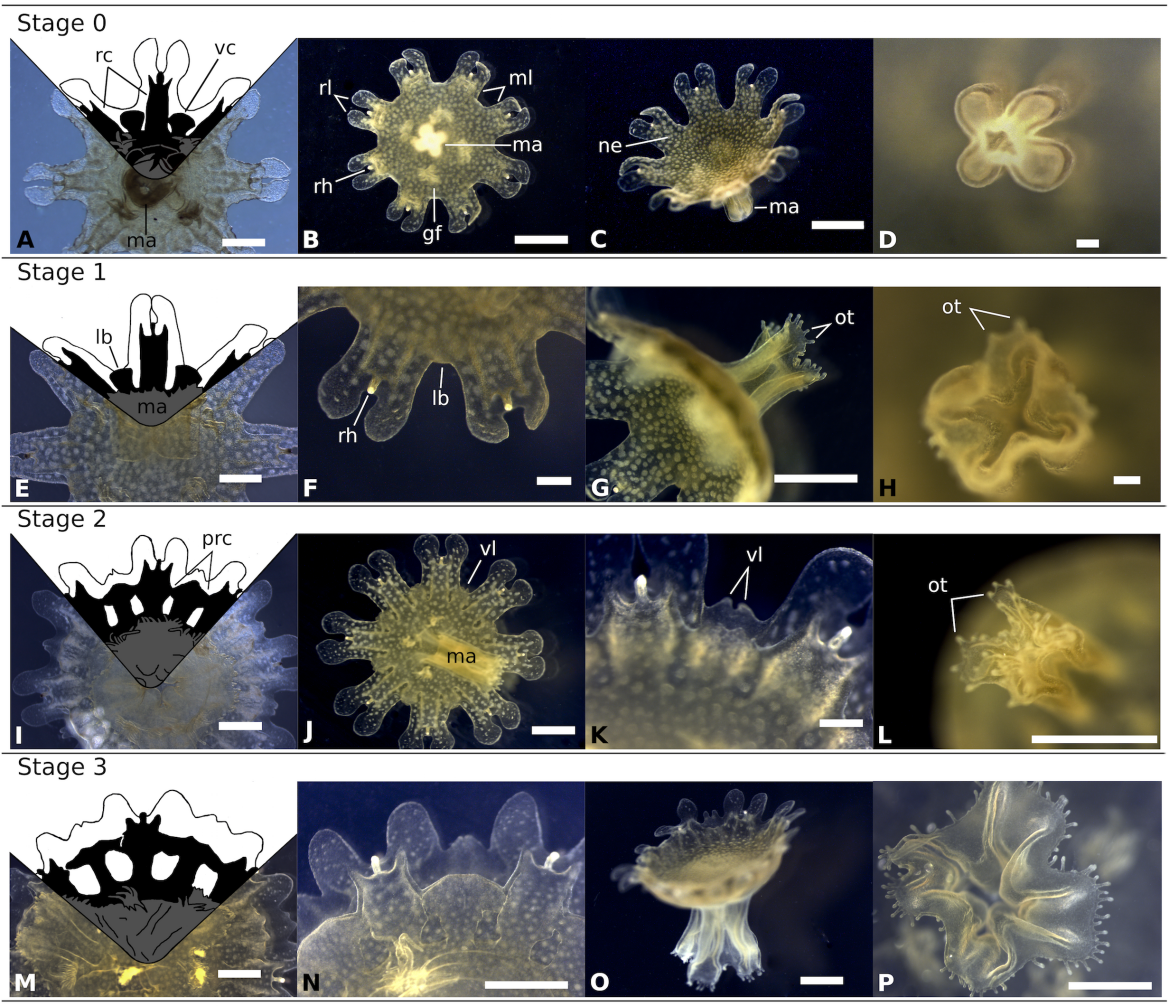
Typical stages in the development of *Rhizostoma luteum* ephyra. **Stage 0 to 3. Stage 0 (A-D):** Newly released ephyra; **(A)** Drawing of gastric system; **(B)** Oral view; **(C)** Aboral view; **(D)** Cross-shaped mouth without oral tentacles; **Stage 1 (E-H):** 48 h post-liberation; **(E)** Drawing of gastric system; **(F)** Enlargement of marginal lappets; **(G)** Side view; **(H)** Cross-shaped mouth with oral tentacles; **Stage 2 (I-L):** 3–4 days post-liberation; **(I)** Drawing of gastric system; **(J)** Oral view; **(K)** Enlargement of marginal lappets; **(L)** Cross-shaped mouth with oral tentacles; **Stage 3 (M-P)**: 5–6 days post-liberation; **(M)** Drawing of gastric system; **(N)** Enlargement of marginal lappets; **(O)** Side view; **(P)** Enlargement of manubrium. *Black* gastric system, *gf* gastric filaments, *lb* lappet bud, *ma* manubrium, *ml* marginal lappets, *ne* nematocyst, *ot* oral tentacles, *prc* primary ring canal, *rc* rhopalar canal, *rh* rhopalium, *rl* rhopalar lappets, *vc* velar canal, *vl* velar lappets. Scale bar 1 mm for all except (D, F, H) 100 μm. Drawings by R. Halbauer. Photos by K. Kienberger.

Newly liberated ephyrae reached between 3.41 to 4.52 mm width from lappet tip to lappet tip when extended (TBD; n = 10; 4.01 ± 0.35 mm) with a mean central disc diameter (CDD) of 2.39 ± 0.22 mm, about 60% of the TBD (see Fig 4 for body proportions and Table 2 for mean values). Typical ephyrae had 8 marginal lappets (Fig 7A–7C), about 17% were irregular ephyrae and mostly with 9 marginal lappets. Using the identification key for young ephyrae defined in [18], each arm contained a pair of bread knife-like lappets (rounded tips) and a single rhopalium, situated between the two lappets. Ephyrae were coloured from light mustard to dark gold with darker nematocyst warts scattered over the exumbrella. The four-lipped (cross-shaped) manubrium was long (Fig 7C and 7D), approx. 45% of the CDD. The gastric system consists of a central stomach and 16 gastric pouches. The 8 rhopalial canals were slightly forked, the tips of which end at the base of the rhopalium, with forked sharp tips (Fig 7A), and 8 velar (adradial) canals were flat rhombical (defined in [18]) and their tips end at the umbrella rim. Each gastric cavity per quadrant had three to seven gastric filaments (Fig 7A and 7B).

**Table 2:** Morphometric measures of *Rhizostoma luteum* ephyra.

Stage/days	*n*	TBD	CDD	TMLL	ML
0 / 0	10	4.01 ± 0.35	2.39 ± 0.22	0.93 ± 0.10	0.71 ± 0.22
1 / 1	7	4.86 ± 0.96	2.71 ± 0.51	1.21 ± 0.23	0.86 ± 0.25
2 / 3–4	7	5.81 ± 0.85	3.69 ± 0.58	1.19 ± 0.19	1.20 ± 0.28
3 / 5–6	7	7.02 ± 0.91	4.80 ± 0.68	1.27 ± 0.16	1.93 ± 0.41
4 / 7–8	7	7.78 ± 1.04	5.91 ± 1.01	1.18 ± 0.21	2.42 ± 0.33
5 / 9–15	7	11.10 ± 2.19	9.38 ± 2.42	1.22 ± 0.16	3.41 ± 0.41
6 / 16–20	6	15.37 ± 2.15	14.41 ± 2.52		
7 / 21–25	6	19.40 ± 2.56	18.30 ± 2.54		

Mean value (mm) and standard deviation of morphometric measures analysed in the 7 ephyra stages of *Rhizostoma luteum* reared in the laboratory. *n* number of individuals measured, *TBD* total body diameter, *CDD* central disc diameter, *TMLL* total marginal lappet length, *ML* manubrium length.

#### Stage 1: 48h post-liberation (Fig 7E–7H)

TBD and ML increased approx. 21% and the marginal lappet elongated 30%. Tiny oral tentacles developed on the distal ends of the manubrium. Between the marginal lappets, the apices of the velar canals expanded outwards and a there were velar lappet bud outgrowths from the gastric canal.

#### Stage 2: 3–4 days post-liberation (Fig 7I–7L)

The manubrium continued to lengthen (approx. 40% since stage 1). Pairs of small velar lappets developed from the velar lappet buds situated between the marginal lappets. The velar canals fused with the rhopalial canals forming a primary ring canal.

#### Stage 3: 5–6 days post-liberation (Fig 7M–7P)

The velar lappet pairs widened and expanded outwards. The manubrium commenced to split into 4 oral arms (approx. length 61% increment since Stage 2). Some connections of the primary ring canal between the velar and rhopalial canals were beginning to separate.

#### Stage 4: 7–8 days post-liberation (Fig 8A–8D)

After one week, the TBD doubled in size and the ML tripled in size. However, the marginal lappet reduced its total length, as the CDD expanded. All connections of the primary ring canal were closed and eliminated.

**Fig 8:**
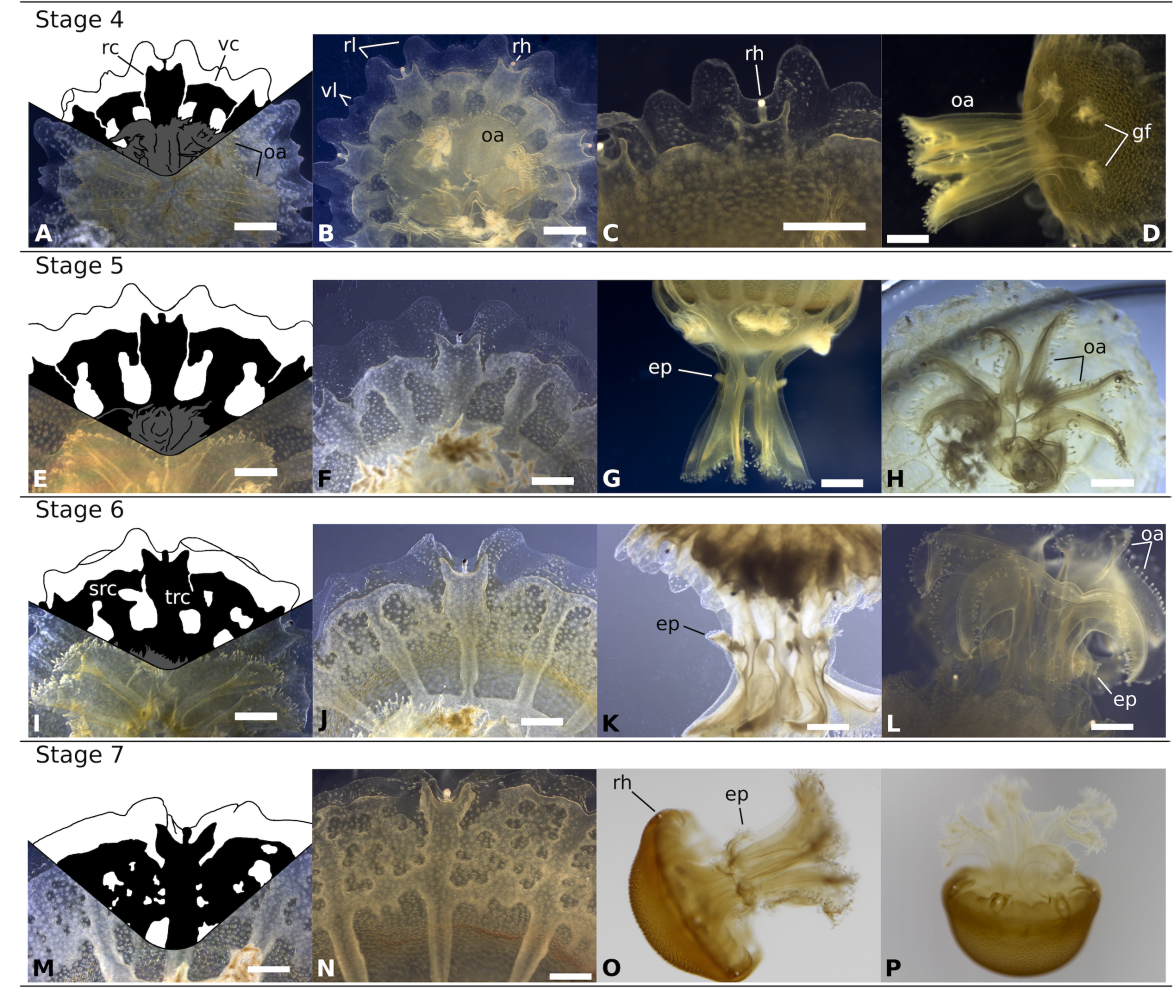
Typical stages in the development of *Rhizostoma luteum* ephyra. **Stages 4 to 7. Stage 4 (A-D):** 7–8 days post-liberation; **(A)** Drawing of gastric system; **(B)** Oral view; **(C)** Enlargement of marginal lappets; **(D)** Side view; **Stage 5 (E-H):** 9–15 days post-liberation; **(E)** Drawing of gastric system; **(F)** Oral view; **(G)** Side view; **(H)** Enlargement of oral arms; **Stage 6 (I-L):** 16–20 days post-liberation; **(I)** Drawing of gastric system; **(J)** Oral view; **(K)** Side view; **(L)** Enlargement of oral arms; **Stage 7 (M-P)**: 21–25 days post-liberation; **(M)** Drawing of gastric system; **(N)** Oral view; **(O,P)** Side view; *Black* gastric system, *ep* epaulette, *gf* gastric filaments, *oa* oral arm, *rc* rhopalar canal, *rh* rhopalium, *rl* rhopalar lappets, *src* secondary ring canal, *trc* third ring canal, *vc* velar canal, *vl* velar lappets. Scale bar 1 mm. Drawings by R. Halbauer. Photos by K. Kienberger: A-N; A.M. Schönemann: O,P.

#### Stage 5: 9–15 days post-liberation (Fig 8E–8H)

The velar lappet extremities extended almost until the rhopalial lappet tips, completing the umbrella. The four oral arms divided, forming eight. Above the oral arms, eight epaulettes buds started to develop on the manubrium. The disconnected side branches of the velar canals grew to a T-like shape.

#### Stage 6: About 16–20 days post-liberation (Fig 8I–8L)

TBD tripled since the liberation of the ephyra and the CDD was 94% of the TBD. The oral arms changed to become J-shaped in lateral view. Small oral tentacles developed on the epaulettes. The velar canal formed a secondary ring canal and fused with the rhopalial canals forming a third ring canal.

#### Stage 7: About 21–25 days post-liberation (Fig 8M–8P)

3–4 weeks after the ephyra released from the strobila, the TBD and CDD increased by about 4.8 and 7.6 times, respectively. The velar canals developed a fine-meshed network. The mesogloea thickened and the flat exumbrella of the young ephyra transformed into a typically bell-shaped medusa (metaephyra). The bell of the metaephyra was entirely of a dark golden yellow colour and the oral arms were slightly lighter in colour. During the first seven stages the ephyra displayed a linear grow. TBD had a mean increase of 0.68 mm per day (n = 126, TBD = 0.68 day + 3.16, R^2^ = 0.90, p < 0.0001). Similarly, the CDD and ML increased linearly with growth (n = 126, CDD = 0.76 day + 0.75, R^2^ = 0.90, p < 0.0001; n = 64, ML = 0.27 day + 0.37, R^2^ = 0.905, p < 0.0001). The raw data file has been included in the Supporting Information (S3 Dataset and S1 Fig).

### Young medusae

**Fig 9:**
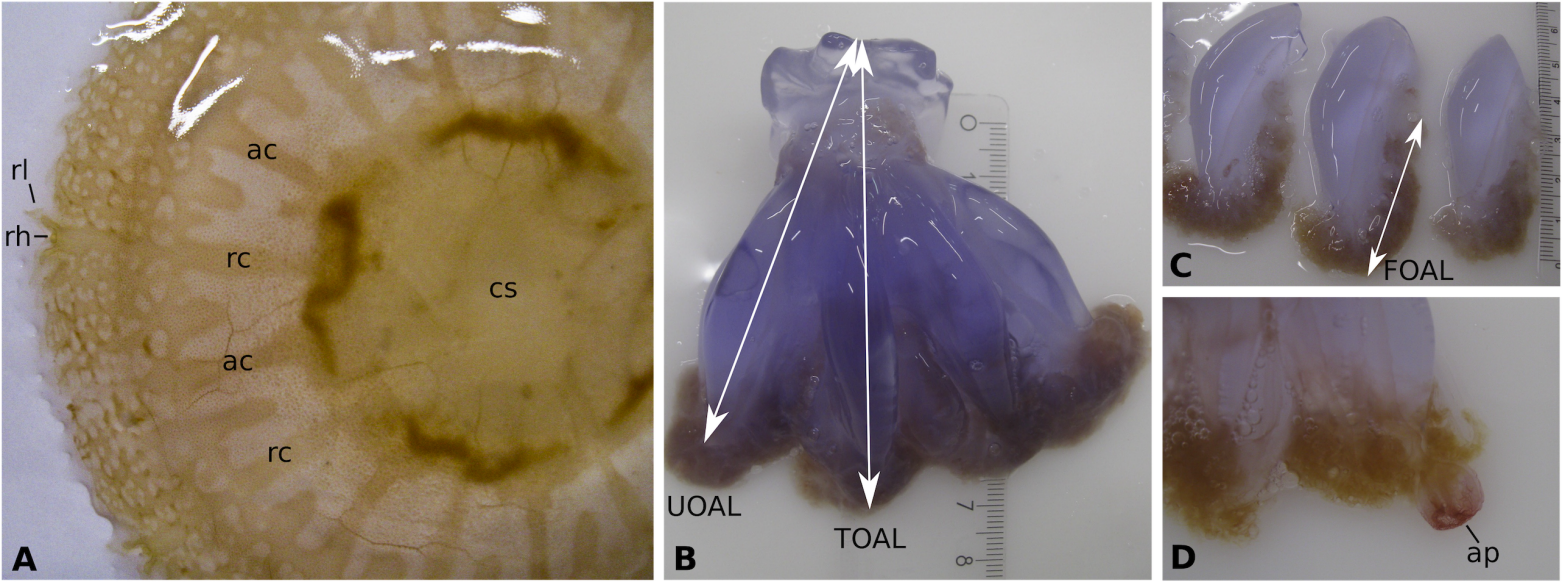
Young medusa *Rhizostoma luteum*: 2-month post-liberation. **(A)** Aboral view, bell diameter 5.8 cm; **(B)** Amputated manubrium; **(C)** Amputated oral arms; **(D)** Oral arm with one terminal appendage, length 0.2 cm; *ac* adradial canal; *ap* appendage, *cs* central stomach; *rc* rhopalar canal; *rl* rhopalar lappets; *rh* rhopalium; *FOAL* frilled oral arms length, *TOAL* total oral arm length, *UOAL* unfrilled oral arm length. Photo by K. Kienberger.

Two months after ephyra release, the young medusa reached a bell diameter of 8.1 to 9.9 cm (n = 5; 8.66 ± 0.72 cm) and a wet weight between 35 to 59 g (n = 5; 44 ± 10 g). The gastrovascular canal system was well developed (Fig 9A). The 16 radial canals were connected to each neighbour, tracing a circular fine-meshed network (anastomosing canals). The anastomosing canals outgrow until the umbrella margin, and being approx. 1/3 of the length of the area between the umbrella margins and the central stomach. The rhopalar lappets were narrow and pointed. Terminal appendages began to develop (Fig 9D). The mean values of morphometric measures that were analysed are summarised in Table 3 and illustrated in Fig 9B and 9C. All young medusae were immature. The raw data file has been included in the Supporting Information (S4 Dataset).

**Table 3:** Morphometric measures of young medusa of *Rhizostoma luteum*.

Time after release	n	Bell size (cm)	Wet weight (g)	TOAL (mm)	UOAL (mm)	FOAL (mm)
2 months	5	8.66 ± 0.72	44 ± 10	3.4 ± 0.6	2.4 ± 0.6	1.3 ± 0.2
3 months	7	13.27 ± 2.26	181 ± 53	7.5 ± 0.6	5.9 ± 0.7	3.6 ± 0.6

Mean values and standard deviation of morphometric measures of young medusa of *Rhizostoma luteum* reared in the laboratory. *n* number of individual measured, *TOAL* total oral arm length, *UOAL* unfrilled oral arms length, *FOAL* frilled oral arms length.

**Fig 10:**
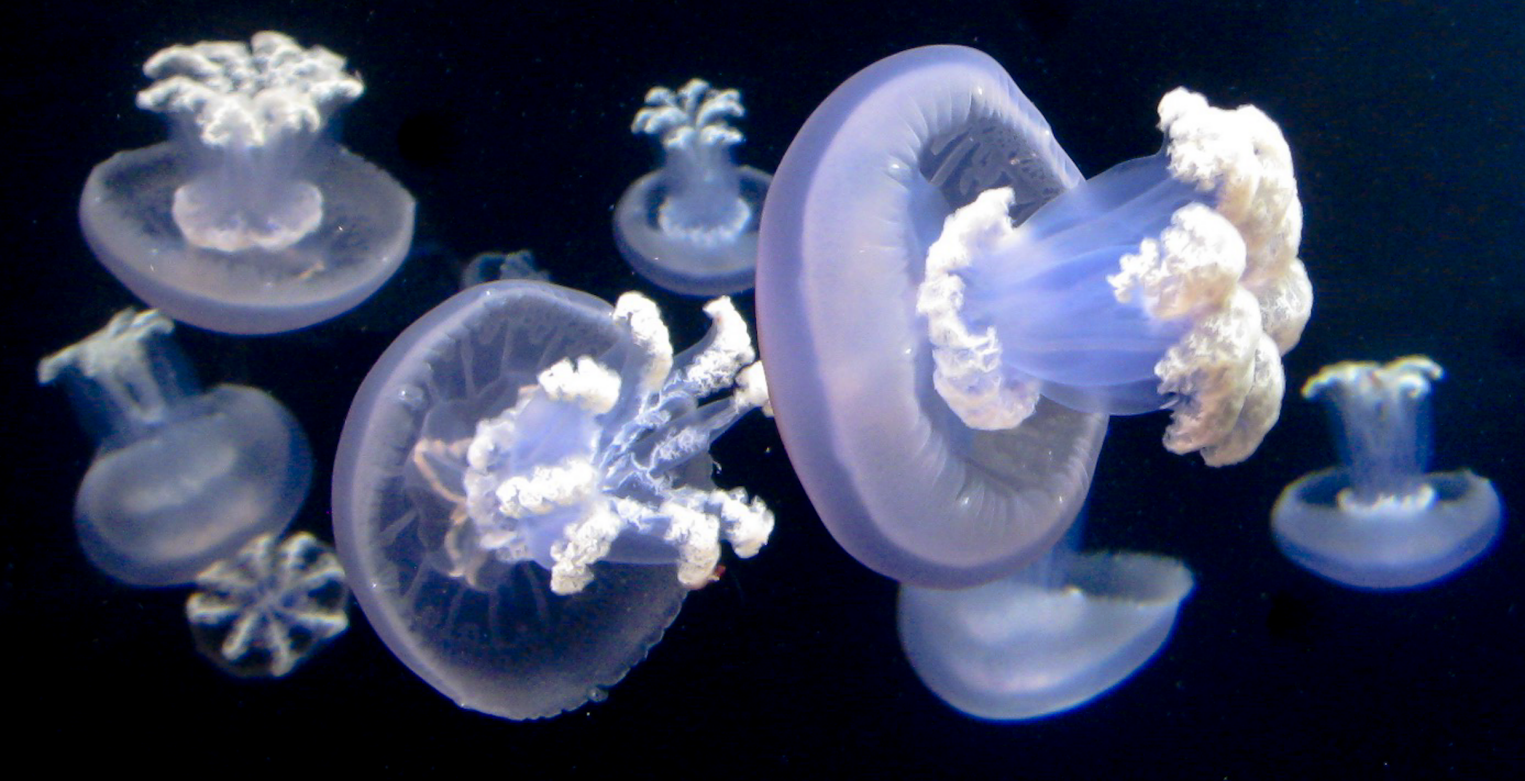
Young medusa of *Rhizostoma luteum* reared in the laboratory. Approximately three- month post-liberation. Photo by K. Kienberger.

**Fig 11:**
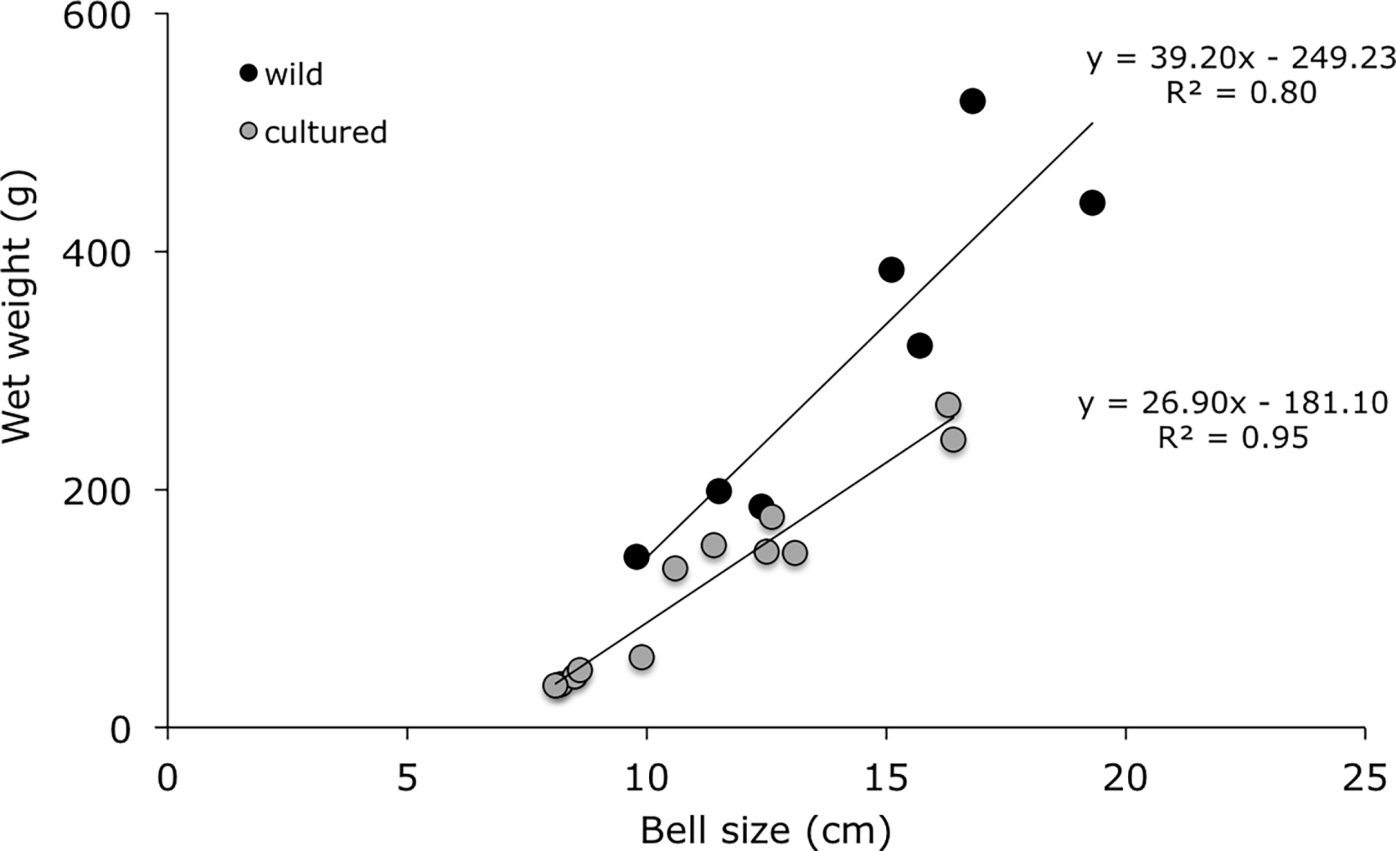
Positive linear relationship between bell diameter and wet weight of young medusa of *Rhizostoma luteum*. Data were compiled from specimens reared in the laboratory (grey dots) and collected in the wild (black dots). Each data point represents a different medusa. Regression lines and their equations are represented.

Three-month post-liberation (Fig 10), the bell diameter reached a mean bell diameter of 13.27 ± 2.26 cm (n = 7) and wet weight of 181 ± 53 g, the largest exemplar having a diameter of 16.4 cm and wet weight of 242 g (see Table 3). The umbrella had a very thick mesogloea, and it was coloured light to dark violet with small reddish-brown warts. Oral arms were also coloured violet with mustard mouth frills. Appendages were light purple-brown when present, the longest measured was 5.5 cm. Less than 2% of the juvenile medusae developed 1 to 3 short terminal appendages, but never developed all 8. In analysing the gonads under a microscope, 4 contained male spermatozoa, and 3 contained oocytes in different development stages and planulae. There was no visible sexual dimorphism in the colour of the gonads. Bell diameter was positively correlated with wet weight (linear regression: n = 12, bell size = 26.904 x—181.1, R^2^ = 0.95, p < 0.001; see Fig 11). The raw data file has been included in the Supporting Information (S4 Dataset).

The young medusae collected in the wild (Table 4) had a bell diameter of 9.8 to 19.3 cm (n = 7; 14.37 ± 3.30 cm) and a wet weight between 143 to 526 g (n = 7; 314 ± 144 g). Having already the typical colouration seen in larger *R*. *luteum*; the umbrella was milky bluish-white with small reddish-brown warts and the oral arms were bluish with mustard mouth frills and with deep purple-brown appendages. 4 specimens had short appendages, but none had all 8 appendages completely developed. The largest appendage was 9.8 cm (bell diameter 16.8 cm). None of the collected jellyfish were sexually mature, as no fertilized eggs, embryos or planulae were present in the gonads. Further, there was a good positive correlation between the bell diameter and wet weight (n = 7, bell size = 39.20 x—249.23, R^2^ = 0.80, p < 00617; see Fig 11).

**Table 4:** Measurements of young medusa of *Rhizostoma luteum* collected in the wild.

Collecting dates (2016)	Bell size (cm)	Wet weight (g)	Number of appendages/ mean length (cm)
2 February	15.7	321	2 / 4.8 ± 0.42
4 February	11.5	198	7 / 4.0 ± 0.70
4 February	16.8	526	7 / 8.17 ± 2.18
4 February	12.4	186	1 / 2.2
4 February	19.3	441	Not present
8 February	9.8	143	Not present
8 February	15.1	384	Not present

## Discussion

*Rhizostoma luteum* has a typical metagenetic scyphozoan life cycle in which benthic scyphopolyps asexually strobilate ephyrae that grow into sexually reproducing medusae. In general, its life cycle is similar to its congeners, with the distinction that *R*. *luteum* is a brooding species and the strobilation type is predominantly monodisc. Brooding is common among the Rhizostomeae medusae [15, 26]. However, to our knowledge, this character is described for the first time in the genus *Rhizostoma*, as the other two species *R*. *pulmo* [27, 28] and *R*. *octopus* [15, 29–31] were defined as non-brooding medusae (see review Table 5).

**Table 5:** Summary of the characters of the planula, scyphistoma, strobilation process and ephyra of the genus *Rhizostoma* described by different authors.

Species	Planulae brooded	Culture conditions (°C)/ salinity/ light cycle	Planula size length/ width (μm)	Settl-ment (days)	Scyphistoma fully developed size range (mm)	Number of tentacles	Scyphistoma hypostome	Asexual reproduction	Strobilation rate (ephyrae per strobila; strobilation type)	Strobilation duration (days)/ Strobilation temperature	Ephyra size after release (mm in diameter)	No. of marginal lappets	Shape of rhopalial lappet	Colour of ephyra	Reference
***R*. *luteum***	✓	17–17.5°C/ 37/ 12:12	126–139; mean 132/ 73–106; mean 84	3–5	1.34–2.5; mean: 1.74	14–16 filiform tentacles	Conspicuous and flexible in all stages	Podocysts	1; monodisc	5-6/ at 17.5°C	3.41–4.52; mean: 4.01	Typical 8, up to 11	Bread knife shaped	Light yellow to light brown	Present study
***R*. *pulmo***	✗	n.d.	500/n.d.	n.d.	12	32	Long and flexible	Polyp buds, swimming buds, podocysts	12–18 segments (no detachment of ephyrae)	No develop-ment of ephyrae/ temperature changes has no effect	No development of ephyrae	No develop-ment of ephyrae	No develop-ment of ephyrae	No develop-ment of ephyrae	[27]
***R*. *pulmo***	n.d.	10–15°C/ 36/ without light	n.d.	n.d.	1.45	16	Long, club shaped	Podocysts, lateral polyp buds	8; polydisc	n.d.	3.19–3.34; mean: 3.27	8	Spade like	Milk trans-parent	[18,32]
***R*. *pulmo***	n.d.	21°C/ 37-38/ 12:12	n.d.	n.d.	0.96–2.15; mean: 1.69	14–18 filiform tentacles, mean: 16	Long	Lateral polyp buds, stolonial polyp buds, podocysts, pedalocysts	8; polydisc	n.d.	2.28–3.93; mean: 3.17	Typical 8, range 5–9	Spade to lancet-shaped	Opaque white	[28]
***R*. *pulmo***	n.d.	14°, 21°, 28°C/ 38-39/ 12:12	n.d.	n.d.	n.d.	n.d.	n.d.	Buds	13.5 at 14°C; 6.8 at 21°C; 8.5 at 28°C; polydisc	40.5/ at 14°C; 12.2/ at 21°C; 13.5/ at 28°C	n.d.	n.d.	n.d.	n.d.	[33]
***R*. *pulmo***	n.d.	10°, 15°, 20°C/34/dark	n.d.	n.d.	n.d.	n.d.	n.d.	Podocysts	n.d.	n.d.	n.d.	n.d.	n.d.	n.d.	[34]
***R*. *octopus***	n.d.	n.d.	100–110	3–5	0.4	10–16	n.d.	Podocysts	n.d.	n.d.	n.d.	8	n.d.	n.d.	[35]
***R*. *octopus***	✗	5–20°C/35/ n.d.	110-150/ 80–90	1–5	2.3	Up to 24	Large and flexible	Podocysts, polyp buds (rare)	1–5; polydisc	18-31/ change from 15–10°, 5–10°or 10–15°	2.7–5.8; mean 4.5	8	n.d.	n.d.	[15,30]
***R*. *octopus***	n.d.	5–10°C/ 36/ without light	n.d.	n.d.	1.9	16–20	Conspicuous and flexible in all stages	Podocysts, lateral polyp buds	5–12; polydisc	n.d.	3.30–5.96; mean: 4.81	8	Bread knife shaped	Milky trans-parent	[12,32]
***R*. *octopus***	✗	10°C/ 35/n.d.	107–148; mean 127/ 76–105; mean 85	2	n.d.	n.d.	Conspicuous and flexible in all stages	Laterally polyp buds, podocysts, longitudinal fission	1–5; polydisc	7-34/ 10°C constant	2.7–5.8; mean: 4.5	8	n.d.	Light yellow to light brown	[31]

*n*.*d*. not documented

The order Rhizostomeae has been divided into two suborders Kolpophorae and Dactyliophorae (including the genus *Rhizostoma*), based on the difference in the development of their gastrovascular network [10]. The development of the canal system in *R*. *luteum* was typical of the suborder Dactyliophorae, forming outwards from the ring canals and having 16 canals connecting to the stomach (see Figs 7–9). Dactyliophorae are mostly non-brooding and they are typically polydisc under optimal conditions (Table 6), except for *Catostylus mosaicus* and *Rhopilema verrilli*, which retain their planulae and usually have monodisc strobilation (reviewed in [15, 26]), identical to *R*. *luteum* (present study). On the other hand, all Kolpophorae species with known life cycles are brooding species and their strobilation type is monodisc. Within Scyphozoa evolution, polydisc strobilation appears to be an ancestral character [36] with monodisc strobilation having arisen in Kolpophorae, *Sanderia malayensis* (reviewed in [37]) and in *R*. *luteum* (present study). Moreover, all Dactyliophorae produce podocysts ([15], *R*. *luteum* present study), they are chitin-covered cysts, which form beneath the pedal discs of the scyphistoma, containing stored nutritional reserves and they can stay dormant for a prolonged period of time (i.e. *Aurelia aurita* cysts survived for 3.2 years [38]). In 2009 Arai wrote, “The earliest literature speculated that podocysts were produced during poor conditions, and that they provided protection against predation or a limited food supply. More recent papers show that podocysts may indeed protect against predation, but the rate of their production is usually positively correlated with the availability of food in otherwise good conditions” [39]. The suggestion by Arai was confirmed by recent studies, showing that the rate of podocyst production is affected by environmental conditions such as temperature, food supply [34], salinity, and dissolved oxygen concentration [38].

**Table 6:** Summary of brooding behaviour and ephyrae per strobila of the suborder Dactyliophorae (Rhizostomeae) as described by different authors.

Family	Species	Brooding behaviour	Ephyrae per strobila	References
**Catostylidae**	*Catostylus mosaicus*	✓	Typically monodisc; up to 5	[40]
**Lychnorhizidae**	*Lychnorhiza lucerna*	✗	Polydisc; 3	[26,41]
**Rhizostomatidae**	*Rhizostoma pulmo*	✗	Polydisc; 12–18	[32]
	*Rhizostoma octopus*	✗	Polydisc; 1–5	[18,29]
	*Rhizostoma luteum*	✓	Monodisc	Present study
	*Rhopilema esculentum*	✗	Polydisc; up to 17	[42]
	*Rhopilema nomandica*	✗	Polydisc; 5–6	[43]
	*Rhopilema verrilli*	✓	Typically monodisc; up to 3	[44,45]
**Stomolophidae**	*Nemopilema nomurai*	✗	Polydisc; 3–7	[46]
	*Stomolophus meleagris*	✗	Polydisc; 1–3 typically 2	[47]

In the present study, the production of podocysts was significantly higher only in week 8 and 11 between the treatments with and without a drop in temperature. After the temperature was set back to 17–17.5 °C, the podocysts were able to rapidly regenerate new scyphistomae and, subsequently, they strobilated and produced ephyrae. Fewer, and only from week 11 onwards, podocysts were produced in the control; no excysting was monitored (see Fig 5). Furthermore, formations of finger-shaped stolons were observed on the stalk (Fig 6D–6F) of fully-grown scyphistomae (16 tentacles). These stolons were merely part of podocyst formation, as the release of a bud-like formation or the formation of tentacles were not observed. Further, in a previous study the rhizostome propagation was defined mostly by a reduced number of asexual scyphistoma-to-scyphistoma modes (mainly podocysts: Type 5 [34, 17]). *Rhizostoma pulmo* asexual reproduction occurred by lateral budding, by means of stolons, and podocysts (Type 1, 3, 5 [28, 17]). Podocyst production was more frequent and occurred without changes in temperature (21 °C temperature, 37–38 salinity, and 12:12 light cycle). The rate of asexual reproduction in *R*. *octopus* was low at all cultivation temperatures, nevertheless, the most frequent mode was the production of podocysts (Type 5 [15, 17]) at a constant temperature of 20 °C (salinity 35). Actually, fewer were formed at a lower temperature. The rate of lateral budding by means of stolons (Type 3 [15, 17]) was low at all cultivation temperatures (less than 1% during a 2-year observation period).

As with other species of the genus, the fully developed scyphistomae of *R*. *luteum* had a conspicuous hypostome and filiform tentacles (Fig 2F). The body proportion ratios of *R*. *luteum* (calyx ~ 45%, stalk ~ 20%, hypostome ~ 35%) correspond to the group of Rhizostomida, as described in [48]. Scyphistoma never lost its filiform tentacles (Fig 2G–2J) during the various phases of strobilation. Furthermore, the large hypostome could be a useful character with which to identify scyphistomae in the field, as the only other Rhizostomeae abundant in the western Mediterranean Sea is *Cotylorhiza tuberculata*, which does not have a significant hypostome [24]. All of the *R*. *luteum* scyphistomae in the present study always survived and were able to feed after the liberation of ephyra. On the other hand, *Cotylorhiza tuberculata* has a higher mortality rate of scyphistoma after strobilation (up to 92% [24]). Under the tested conditions, the predominant type of strobilation observed in *R*. *luteum* was monodisc, being therefore a distinctive feature of the species compared to its sibling species, which usually have polydisc strobilation (Table 4) under optimal conditions. *R*. *luteum* scyphistomae strobilated repeatedly in both treatments and produced viable ephyrae. *R*. *pulmo* scyphistomae strobilated spontaneously also under constant temperature in springtime (21 °C temperature, 37–38 salinity and 12:12 light cycle [28]). However, *R*. *octopus* did not produce ephyrae without a temperature change (15 to 10 °C; 5 to 10 °C; 10 to 15 °C [15]). Ephyrae of *R*. *luteum* generally developed into an 8-rayed type typical of scyphozoan taxa with 16 bread knife-like rhopalial lappets, like *R*. *octopus*, though, *R*. *pulmo* has round spatula-like rhopalial lappets [18]. It took the ephyra approximately 21 days to develop until stage 7 (TBD; n = 6; 19.40 ± 2.54 cm), increasing 4.8 times its total body diameter since its liberation, and having a daily growth rate of 0.68 mm; in comparison *Cotylorhiza tuberculata* had a grow rate of 0.08 mm per day [49]. In general, the development of the ephyra was very similar to its congeners, apart from the important size difference that was already visible in the first week after liberation of the ephyra (*R*. *luteum* (mm): 1w: 7.78 ±1.04, 2w: 11.10 ± 2.19, 3w: 19.4 ± 2.56 (present study); *R*. *octopus* (mm): 1w: 5.8 ± 0.8; 2w: 7.7 ± 0.9; 3w: 9.5 ± 1.3 [15]). At three-months post-liberation, the medusae become mature and they were 5.3 times larger than *R*. *octopus* (2–3 cm [15]; *R*. *luteum*: mean TBD 13.27 cm, present study). However, as soon as the *R*. *luteum* in the present reached the mature state, they did not grow larger. They even started to decrease in size and decomposed completely. It is the belief of the present authors that the significant size difference is due to the consecutive large kreisels that were used to rear the jellyfish. Nonetheless, none of the young medusae showed any sexual dimorphism in the colour of the gonads. Mature *R*. *octopus* collected in the wild, had a sexual dimorphism in the colour of the gonads visible through the bell; female gonads were brown-coloured and contained brown, ripe eggs, and the male gonads were whitish-blue [15]. Moreover, as all of the young medusae that were collected *in situ* had a very similar bell diameter; it can be assumed that they belong to one cohort. This seems to agree with the fact that in temperate coastal and shelf ecosystems, the majority of jellyfish populations consist of single cohorts growing and maturing synchronously, with sexually mature females with planula larvae that are present for between 1 and 5 months, reviewed in [50]. Notwithstanding, in the present study on three occasions mature medusa were collected in the wild, which was in April, May and October (Kienberger, unpublished data). It is interesting to note that there was a significant colour difference between the young medusae that were reared in laboratory conditions and the wild medusae (see Figs 1 and 10). Cultivated medusae having a violet umbrella and oral arms are different to those collected in the wild, which are milky bluish-white in colour. Colouration differences have been seen in the order Rhizostomeae as reared in the laboratory (e.g. *Mastigias papua*, *Phyllorhiza punctata* and *Cotylorhiza tuberculata*; Bartsch & Halbauer, pers. observ.).

Our results suggest that a temperature shock does not have an effect on the mean amount of scyphistoma. However, scyphistomae strobilated and developed podocysts earlier in the treatment with a temperature shock (week 8) than in the control (week 11), see Fig 5. Since the scyphistoma phase of *R*. *luteum* seems highly resistant to fast falls in temperature, a strong episode of upwelling (decreasing sea surface temperature), a usual feature of some regions of the east Atlantic where these jellyfish have been reported, would most likely trigger the production of podocyst and might start the strobilation process. Furthermore, in the hypothetical case, adult medusae would enter the lagoon of the Mar Menor, the scyphistomae phase could survive a severe winter condition (with temperatures as low as that tested in the present study) while, on the contrary, other species such as *Cotylorhiza tuberculata* scyphistomae will not survive [24]. To our knowledge, no data are available for *R*. *pulmo*, which is the other dominant species in the lagoon.

It is suggested in [51] that there are taxonomically and phylogenetically characteristics predisposing some medusozoans to occur *en masse*. These include character states and character complexes related to podocyst formation, strobilation, extensive canal systems, large size, rapid growth rate, elevated Reynolds number, oral arms, lack of photosymbiosis and shallow-water habitat. As all these adaptations are present in *R*. *luteum* and it may predispose it to occur *en masse*. However, to our knowledge, *R*. *luteum* seems does not occur *en masse* at the present, as most of the reported sightings in the last two decades are of individuals or on occasions a few individuals. We know of only one beaching episode on 12 June 2012, where a total of 24 individuals were stranded on a single beach in Doñana National Park (SW Iberian Peninsula, Spain), and that report is the sole example in our dataset ([6, 7] Kienberger, unpublished data), which had more than 10 individuals. On the other hand, *R*. *pulmo* and *R*. *octopus*, reviewed in [52], have been reported to be important blooming coastal species in recent years, showing important inter-annual fluctuations in their population density. Comparing the synapomorphic characteristics related to the ability to form blooms, we believe that *R*. *luteum* may have sufficient pre-requisites to form blooms, when environmental circumstances change, all supposing that *R*. *luteum* would be able to take advantage of those new conditions. Changes in environmental conditions can be driven by a broad variety of diverse causes of different temporal and spatial scales of variability, independently of their sources (natural or antropogenic). The next step for further research would be to evaluate the *R*. *luteum* capacity to form blooms and also to disentangle its response to environmental conditions.

## Conclusion

As expected, the life cycle of *Rhizostoma luteum* in the present study was similar to that described for its congeners, with the exception of two features: first, the female *R*. *luteum* is a brooding species; second, under the conditions that were tested, the predominant type of strobilation observed was monodisc. As polydisc strobilation is likely an ancestral condition, monodisc strobilation in *R*. *luteum* may have arisen for the first time in the genus *Rhizostoma*. Nevertheless, further studies are needed in order to check whether, under different environmental conditions, this species is able to turn to the polydisc strobilation type as with its congeners. Furthermore, being evolutionarily predisposed to form problematic blooms if environmental conditions permit, it is important to disentangle the environmental control, such as the tolerance limits and optimal conditions for survival and asexual reproduction of early life stages of this jellyfish.

## Supporting information

S1 Dataset Settlement preferences of *Rhizostoma luteum* planulae. *BOTT* bottom of the glass flasks: *GS* glass slides; *LAT* sides of glass flasks.
(PDF)

S2 Dataset *Rhizostoma luteum* scyphistoma, podocyst and ephyra count. Variable list: Initial (before temperature drop), Time (Weeks 8, 11 and 16).
(PDF)

S3 Dataset Morphometric measures of *Rhizostoma luteum* ephyra. *TBD* total body diameter, *CDD* central disc diameter, *TMLL* total marginal lappet length, *ML* manubrium length.
(PDF)

S4 Dataset Morphological measures of young medusa *Rhizostoma luteum*. *TOAL* total oral arm length, *UOAL* unfrilled oral arms length, *FOAL* frilled oral arms length.
(PDF)

S1 Fig Linear regression between time (days) and morphometric measures of *Rhizostoma luteum* ephyra. *TBD* total body diameter, *CDD* central disc diameter, *TMLL* total marginal lappet length, *ML* manubrium length.
(PDF)
