# Supplementary material for: First description of the life cycle of the jellyfish Rhizostoma luteum (Scyphozoa: Rhizostomeae)
Source: PLoS One. 2018 Aug 22;13(8):e0202093. doi: 10.1371/journal.pone.0202093 (PMC6104977; doi:10.1371/journal.pone.0202093)
Supplement: S1 Dataset — BOTT bottom of the glass flasks: GS glass slides; LAT sides of glass flasks. (PDF) [file pone.0202093.s001.pdf]

Flask N°	Location	N° Scyphistoma
1	BOTT	3
1	LAT	2
1	LAT	1
1	LAT	0
2	BOTT	0
2	BOTT	0
2	BOTT	1
2	LAT	3
2	LAT	0
2	LAT	1
2	GS	0
2	BOTT	2
3	LAT	10
3	LAT	4
3	GS	1
4	BOTT	2
4	BOTT	2
4	BOTT	1
4	GS	0
4	BOTT	1
