# Supplementary material for: First description of the life cycle of the jellyfish Rhizostoma luteum (Scyphozoa: Rhizostomeae)
Source: PLoS One. 2018 Aug 22;13(8):e0202093. doi: 10.1371/journal.pone.0202093 (PMC6104977; doi:10.1371/journal.pone.0202093)
Supplement: S2 Dataset — Variable list: Initial (before temperature drop), Time (Weeks 8, 11 and 16). (PDF) [file pone.0202093.s002.pdf]

Flask N°	#1			#2			#3			#4		
	Scyphistoma	Podocyst	Ephyra	Scyphistoma	Podocyst	Ephyra	Scyphistoma	Podocyst	Ephyra	Scyphistoma	Podocyst	Ephyra
Initial	9			3			8			11		
8 weeks	3	32	0	8	34	3	11	72	11	4	10	1
11 weeks	5	38	1	9	37	6	13	52	19	5	13	1
16 weeks	6	45	4	7	36	12	15	na	30	6	16	3

Control	#1			#2			#3		
	Scyphistoma	Podocyst	Ephyra	Scyphistoma	Podocyst	Ephyra	Scyphistoma	Podocyst	Ephyra
Initial	4			2			2		
8 weeks	7	0	0	7	0	0	3	0	0
11 weeks	7	2	0	9	8	1	5	4	1
16 weeks	6	8	6	7	8	5	6	8	5
