# Supplementary material for: First description of the life cycle of the jellyfish Rhizostoma luteum (Scyphozoa: Rhizostomeae)
Source: PLoS One. 2018 Aug 22;13(8):e0202093. doi: 10.1371/journal.pone.0202093 (PMC6104977; doi:10.1371/journal.pone.0202093)
Supplement: S3 Dataset — TBD total body diameter, CDD central disc diameter, TMLL total marginal lappet length, ML manubrium length. (PDF) [file pone.0202093.s003.pdf]

| Days | TBD | CDD | TMLL | ML |
|---|---|---|---|---|
| 1 | 4436 | 2461 | 1165 | 1045 |
| 1 | 3195 | 1758 | 777 | na |
| 1 | 2897 | 1707 | 609 | 489 |
| 1 | 3893 | 1927 | 1081 | na |
| 1 | 4640 | 2360 | 1133 | 766 |
| 1 | 4447 | 2423 | 1226 | 548 |
| 1 | 3573 | 2143 | 808 | 714 |
| 2 | 5950 | 3243 | 1374 | 1176 |
| 2 | 3661 | 2107 | 868 | na |
| 2 | 3380 | 1952 | 881 | 523 |
| 2 | 5452 | 2841 | 1417 | 933 |
| 2 | 5093 | 2651 | 1308 | 853 |
| 2 | 5308 | 2985 | 1317 | 648 |
| 2 | 5187 | 3218 | 1277 | 1056 |
| 3 | 5975 | 3604 | 1293 | 1196 |
| 3 | 4563 | 2656 | 1074 | 593 |
| 3 | 4556 | 2865 | 823 | 885 |
| 3 | 5493 | 3622 | 1111 | 1253 |
| 3 | 5987 | 3503 | 1500 | 1233 |
| 3 | 6164 | 3869 | 1207 | 1300 |
| 3 | 5734 | 3517 | 1195 | 924 |
| 4 | 6750 | 4120 | 1212 | 1391 |
| 4 | 4859 | 2911 | 1028 | 1302 |
| 4 | 4889 | 3654 | 1121 | 1119 |
| 4 | 6682 | 4466 | 1083 | 1188 |
| 4 | 6852 | 4367 | 1526 | 1663 |
| 4 | 7011 | 4490 | 1439 | 1491 |
| 4 | 5828 | 3991 | 1107 | na |
| 5 | 7360 | 5057 | 1316 | 1908 |
| 5 | 5193 | 3447 | 930 | 1737 |
| 5 | 5848 | 3989 | na | 1324 |
| 5 | 7469 | 5117 | 1119 | 1791 |
| 5 | 7170 | 4582 | 1468 | 1257 |
| 5 | 8129 | 5407 | 1391 | na |
| 5 | 6721 | 4502 | 1271 | na |
| 6 | 7530 | 5469 | 1113 | 1965 |
| 6 | 5780 | 4051 | 1355 | 2534 |
| 6 | 6437 | 4365 | 1174 | 1565 |
| 6 | 8162 | 5760 | na | 2262 |
| 6 | 7197 | 4707 | 1425 | 2449 |
| 6 | 7779 | 5174 | 1314 | 2247 |

| | | | | |
|---|---|---|---|---|
| 6 | 7506 | 5511 | 1393 | 2062 |
| 7 | 7728 | 6159 | 1096 | 3131 |
| 7 | 5693 | 4107 | 855 | 2304 |
| 7 | 6513 | 4492 | 1150 | 2130 |
| 7 | 7885 | 6268 | 797 | 2124 |
| 7 | 7438 | 5788 | 1060 | 2745 |
| 7 | 8000 | 6784 | na | 2479 |
| 7 | 8074 | 5802 | 1516 | 2434 |
| 8 | 8800 | 6644 | na | 2337 |
| 8 | 6367 | 4443 | 1441 | 2451 |
| 8 | 7344 | 5386 | 1234 | 2108 |
| 8 | 9000 | 7100 | 1334 | 1933 |
| 8 | 8049 | 5765 | 1309 | 2520 |
| 8 | 9000 | 7217 | 1212 | na |
| 8 | 9000 | 6794 | 1174 | 2819 |
| 9 | 11000 | 8000 | 1484 | 3251 |
| 9 | 7583 | 5674 | 1160 | 2953 |
| 9 | 6784 | 5347 | na | na |
| 9 | 9500 | 7500 | 1151 | na |
| 9 | 8385 | 6158 | 1260 | 2931 |
| 9 | 9400 | 7085 | na | na |
| 9 | 9100 | 7000 | 1250 | na |
| 10 | 11500 | 9000 | 1484 | 3401 |
| 10 | 8000 | 6129 | na | na |
| 10 | 7537 | 5499 | 1027 | 2418 |
| 10 | 10500 | 10000 | | na |
| 10 | 9500 | 7000 | 1533 | 3470 |
| 10 | 10500 | 9500 | 1392 | na |
| 11 | 12500 | 10500 | 1250 | na |
| 11 | 8000 | 6200 | 1224 | 3717 |
| 11 | 12000 | 12500 | 1179 | 3518 |
| 11 | 9500 | 7000 | 1178 | 3555 |
| 11 | 11500 | 10000 | 1253 | na |
| 11 | 12000 | 11500 | na | na |
| 12 | 13000 | 11500 | 1188 | 3419 |
| 12 | 9000 | 7500 | 1115 | 3374 |
| 12 | 12500 | 11500 | 802 | |
| 12 | 10000 | 8500 | 1250 | 3652 |
| 12 | 12500 | 11000 | na | na |
| 13 | 14000 | 12000 | 1020 | na |
| 13 | 11000 | 9000 | 1083 | na |
| 13 | 13000 | 12000 | na | na |

| | | | | |
|---|---|---|---|---|
| 13 | 12000 | 9000 | 1355 | na |
| 13 | 13000 | 11000 | na | na |
| 14 | 11500 | 10000 | 1279 | 4133 |
| 14 | 14000 | 13000 | 1085 | 3850 |
| 14 | 11500 | 10000 | 1214 | 3532 |
| 14 | 13500 | 12000 | | |
| 15 | 12000 | 9000 | | |
| 15 | 16000 | 13500 | | |
| 15 | 14000 | 12000 | | |
| 15 | 13500 | 13000 | | |
| 16 | 13000 | 11500 | | |
| 16 | 11000 | 10000 | | |
| 16 | 16500 | 15000 | | |
| 16 | 14000 | 12000 | | |
| 16 | 14000 | 12500 | | |
| 17 | 13500 | 12000 | | |
| 17 | 12500 | 11500 | | |
| 17 | 16500 | 15000 | | |
| 17 | 15000 | 13000 | | |
| 17 | 15000 | 15500 | | |
| 18 | 14000 | 13000 | | |
| 18 | 12500 | 12000 | | |
| 18 | 16500 | 16000 | | |
| 18 | 15000 | 13000 | | |
| 18 | 16500 | 16000 | | |
| 18 | 17000 | 18000 | | |
| 19 | 15000 | 14000 | | |
| 19 | 14000 | 13000 | | |
| 19 | 18000 | 17500 | | |
| 19 | 15000 | 13000 | | |
| 19 | 18500 | 16000 | | |
| 19 | 19000 | 19000 | | |
| 20 | 15500 | 14500 | | |
| 20 | 14500 | 14000 | | |
| 20 | 18000 | 18000 | | |
| 20 | 15000 | 14000 | | |
| 20 | 20000 | 20000 | | |
| 21 | 15500 | 14500 | | |
| 21 | 14000 | 14000 | | |
| 21 | 20500 | 20000 | | |
| 21 | 18000 | 16500 | | |
| 21 | 20000 | 20000 | | |

| | | |
|---|---|---|
| 21 | 20000 | 20000 |
