# Supplementary material for: First description of the life cycle of the jellyfish Rhizostoma luteum (Scyphozoa: Rhizostomeae)
Source: PLoS One. 2018 Aug 22;13(8):e0202093. doi: 10.1371/journal.pone.0202093 (PMC6104977; doi:10.1371/journal.pone.0202093)
Supplement: S4 Dataset — TOAL total oral arm length, UOAL unfrilled oral arms length, FOAL frilled oral arms length. (PDF) [file pone.0202093.s004.pdf]

Days	Wet Weight (g)	Bell size (cm)	TOAL (mm)	UOAL (mm)	FOAL (mm)
56	58.9	9.9	45	33	11
56	36.2	8.2	31	24	14.83
56	43.1	8.5	31	17	11.2
56	47.9	8.6	35	24	16.5
56	34.7	8.1	29	20	12.4
103	270.7	16.3	84	54	32
103	133.2	10.6	67	50	37.2
103	177.2	12.6	76	66	39.75
109	241.8	16.4	na	na	45
109	153.2	11.4	72	56	38.33
109	147.2	12.5	78	66	34.75
109	147.1	13.1	75	64	26.6

Bell size	2 month	3 month
mean	8.66	13.27
max	9.90	16.40
min	8.10	10.60
SD	0.72	2.26

Wet Weight	2 month	3 month
mean	44.16	181.49
max	58.90	270.70
min	34.70	133.20
SD	9.81	53.39
