# Supplementary figures and images for: First description of the life cycle of the jellyfish Rhizostoma luteum (Scyphozoa: Rhizostomeae)
Source: PLoS One. 2018 Aug 22;13(8):e0202093. doi: 10.1371/journal.pone.0202093 (PMC6104977; doi:10.1371/journal.pone.0202093)

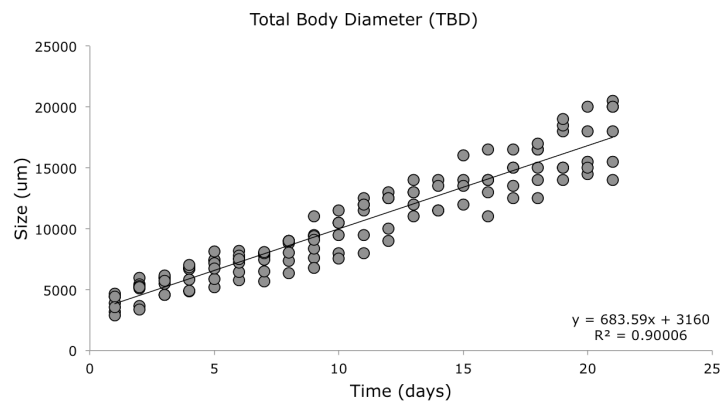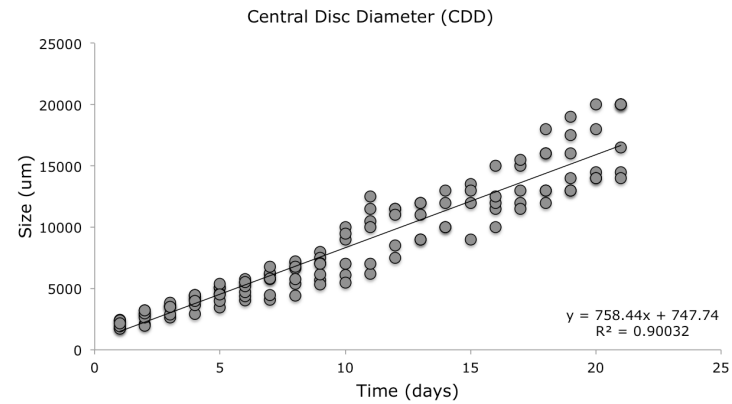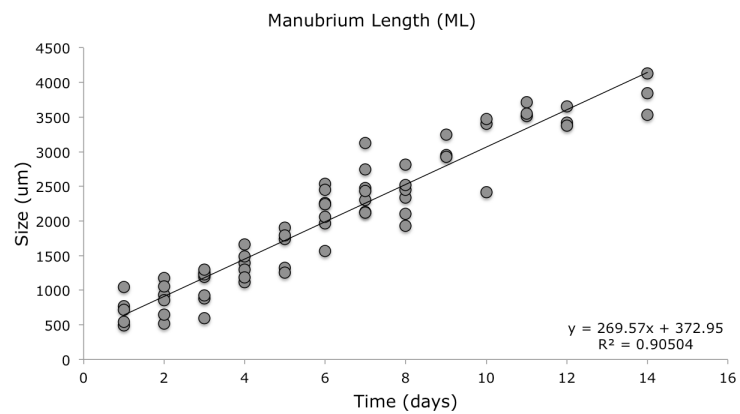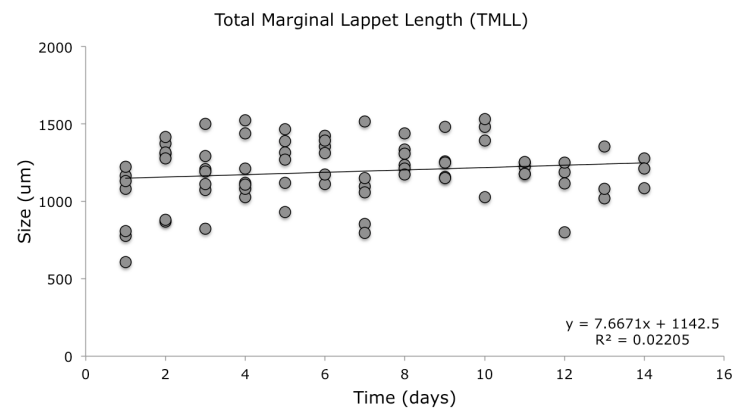

Supplement: S1 Fig — TBD total body diameter, CDD central disc diameter, TMLL total marginal lappet length, ML manubrium length. (PDF) [file pone.0202093.s005.pdf]
